# The repetitive component of the sunflower genome as shown by different procedures for assembling next generation sequencing reads

**DOI:** 10.1186/1471-2164-14-686

**Published:** 2013-10-06

**Authors:** Lucia Natali, Rosa Maria Cossu, Elena Barghini, Tommaso Giordani, Matteo Buti, Flavia Mascagni, Michele Morgante, Navdeep Gill, Nolan C Kane, Loren Rieseberg, Andrea Cavallini

**Affiliations:** 1 Department of Agricultural, Food, and Environmental Sciences, University of Pisa, Via del Borghetto 80, I-56124 Pisa, Italy; 2 Department of Crop and Environmental Sciences, University of Udine, Via delle Scienze, Udine, Italy; 3 The Biodiversity Research Centre and Department of Botany, 3529–6270 University Blvd, University of British Columbia, Vancouver, BC V6T 1Z4, Canada; 4 Ecology and Evolutionary Biology Department, UCB 334, University of Colorado, Boulder, CO 80309, USA

**Keywords:** Genome structure, Next Generation Sequencing, Repetitive DNA, Retrotransposon, Sunflower

## Abstract

**Background:**

Next generation sequencing provides a powerful tool to study genome structure in species whose genomes are far from being completely sequenced. In this work we describe and compare different computational approaches to evaluate the repetitive component of the genome of sunflower, by using medium/low coverage Illumina or 454 libraries.

**Results:**

By varying sequencing technology (Illumina or 454), coverage (0.55 x-1.25 x), assemblers and assembly procedures, six different genomic databases were produced. The annotation of these databases showed that they were composed of different proportions of repetitive DNA families. The final assembly of the sequences belonging to the six databases produced a whole genome set of 283,800 contigs. The redundancy of each contig was estimated by mapping the whole genome set with a large Illumina read set and measuring the number of matched Illumina reads. The repetitive component amounted to 81% of the sunflower genome, that is composed mainly of numerous families of *Gypsy* and *Copia* retrotransposons. Also many families of non autonomous retrotransposons and DNA transposons (especially of the Helitron superfamily) were identified.

**Conclusions:**

The results substantially matched those previously obtained by using a Sanger-sequenced shotgun library and a standard 454 whole-genome-shotgun approach, indicating the reliability of the proposed procedures also for other species. The repetitive sequences were collected to produce a database, SUNREP, that will be useful for the annotation of the sunflower genome sequence and for studying the genome evolution in dicotyledons.

## Background

Eukaryotic species show considerable variation in genome size. This is especially true in higher plants, whose genome size (1C) ranges from 63 Mbp in *Genlisea margaretae* to 150 Gbp in *Paris japonica*[1,2]. Such differences have evolved mainly because of two processes: polyploidy and DNA amplification of transposons and related sequences. In eukaryotic genomes, the latter process has resulted in the accumulation of many repeated sequences – sequences that are similar or identical to sequences elsewhere in the genome, but whose number of copies is much higher than that possibly achieved through polyploidization. Differences in genome size among species largely depend on the size of this repetitive fraction. In fact, large genomes are filled with repetitive sequences, especially in plants [3]. Some repeats appear to be non-functional, whereas others have played key roles in the evolution of species [4]. For example, the mutagenic action of transposons provides substantial increases in genetic variability [3]. Transposons also create novel functions, and alter the regulatory patterns of genes, resulting in phenotypic variation [5,6].

The advent of next generation sequencing (NGS) represents a major advance for genetical and biological research, producing millions of genomic sequences at ever increasing speed and decreasing cost. Dozens of Gigabases of data can be sequenced in a week for the same cost as a few hundred kilobases of Sanger sequence ([7], updated at http://www.molecularecologist.com/next-gen-fieldguide-2013/). NGS technology has offered the opportunity to acquire genome-scale data for any organism [8-10].

In either reference guided or *de novo* assembly of NGS reads, a major computational task is to manage 'multi-reads', i.e. those reads that map to multiple locations and/or contain highly repeated k-mers [11,12]. An algorithm for reference-guided assembly has three choices [13]: 1) to ignore (hence discard) all multi-reads; 2) to perform the best match approach, in which only the best alignment is reported or, if equally good best match alignments occur, one at random or all of them are reported; 3) to report all alignments up to a maximum number. The first strategy restricts the analysis to unique regions in the genome, by discarding all repeats and limiting discovery of some biologically important variants. The other two strategies enable analyses of repetitive regions, with the best match approach providing a reasonable estimate of coverage [12] and reporting all possible alignments to avoid erroneous choices about read placement.

*De novo* assemblers belong to one of two classes, overlap-based and de Bruijn graph assemblers, that each create different types of graphs from the read data. The sequence assembly is then reconstructed by algorithms that traverse the graphs. Repeats cause branches in these graphs [14] and assemblers, making a guess as to which branch to follow, can create false joins and erroneous copy numbers. In a more conservative approach, the assembler breaks graphs at these branch points, producing an accurate but fragmented assembly.

The most common error of an assembler is the production of a chimaera by joining two repeats that are not close in the genome. To resolve chimaeras the first and most important tool is the use of paired-end reads. Because the distance between the paired reads is known, an assembler can use both the expected distance and the orientation of the reads to reconstruct the correct sequence.

Another strategy for handling repeats is to perform statistics on the depth of coverage for each contig. These statistics cannot show exactly how to assemble every repeat, but they do identify the repeats themselves. The assumption is that if a genome is sequenced, for example, to 50x coverage, the genome should be uniformly covered. This means that most contigs should also be covered at 50x. By contrast, a repetitive region will show deeper coverage, thereby allowing the algorithm to identify it as a repeat. In de novo sequencing of a genome, repeats are usually assembled after the assembly of unique regions, and assemblers use multiple paired-ends to link a repetitive contig to a unique one. However, when the objective of research is not to obtain a complete genome but merely single sequence families, the problem of assembling contigs into more extended ones is less stringent. In addition, in the case of repetitive DNA families, lower coverage can reduce the occurrence of multi-reads and hence can improve repeat assembly into contigs and repeat identification and reconstruction [15-18].

The sunflower genome is large (around 3,500 Mbp, [19]). The repetitive component has been recently characterized using a Sanger-sequenced small insert library [20]. This library provided a first set of sequences (1,638) that were used to analyze the composition of sunflower genome in terms of types and abundance of repetitive elements. The fraction of repetitive sequences amounted to 62% of the sequences, while the putative functional genes accounted for 4%. The largest component of the repetitive fraction was represented by long terminal repeat (LTR) retrotransposons, especially of the *Gypsy* superfamily. Class II transposable elements were barely represented in that library.

A larger effort to characterize the repetitive component of the sunflower genome was then made by analyzing approximately 25% of the genome from 454 random sequence reads [21]. In this study, the sunflower genome was shown to be composed of over 81% transposable elements, 77% of which were long terminal repeat (LTR) retrotransposons.

The retrotransposon component of the sunflower genome was also analysed in detail by assembling and analyzing bacterial artificial chromosome (BAC) clones [21,22]. Buti et al. [22] analysed 3 BAC clones, identifying 18 full-length and 6 incomplete LTR retrotransposons. Among LTR-retrotransposons, non-autonomous elements (the so-called LARDs [23,24]), which do not carry any protein-encoding sequence, were discovered for the first time in sunflower. The insertion time of intact retroelements was measured, based on the divergence of sister LTRs. All isolated elements were inserted relatively recently, especially those belonging to the *Gypsy* superfamily.

These results were confirmed and extended by Staton et al. [21]. The LTR retrotransposon fraction was shown to be composed in large part by chromodomain-containing *Gypsy* LTR retrotransposons. The authors showed that there is a bias in the efficiency of homologous recombination in removing LTR retrotransposon DNA, and provided insight into the mechanisms associated with the composition of the transposable element fraction in the sunflower genome. They also showed that most intact LTR retrotransposons have likely inserted since the origin of this species, providing further evidence that biased LTR retrotransposon activity has played a major role in shaping the DNA landscape of the sunflower genome.

In other studies, retrotransposons of the sunflower have been shown to be conserved within the *Helianthus* genus [25] and transcriptionally active [20,26-28]. Fluorescent *in situ* hybridization studies have suggested that the *Gypsy* and *Copia* superfamilies are most frequent in the heterochromatic regions close to centromeres and telomeres, respectively [29-31]. The genomic organization of *Gypsy* elements is conserved also in hybrid sunflower species derived from the common sunflower, despite large amplification of these elements in the genome of such species [31-33].

The aim of the present study is to verify the suitability of using different approaches of *de novo* assembling sequence reads obtained by NGS procedures (Illumina and 454) to gain a comprehensive characterization of the repetitive component of a plant species (*Helianthus annuus*), whose large-sized genome is being sequenced thanks to the efforts of an international sequencing consortium [21,34]. The repeat structure of the sunflower genome obtained in this study is validated by comparison with those obtained using a sunflower Sanger-sequenced small insert library [20], Sanger- or 454-sequenced sunflower BAC clones [21,22], and sunflower *de novo* assembled 454 reads [21].

Besides developing further resources needed to sequence the sunflower genome, this study highlights the extent to which the repetitive portion of a plant genome can be characterized using NGS, and describes the utility and concerns raised by NGS methods of surveying such sequences.

## Results

### Comparison of different assembled sequence sets

**Figure 1:**
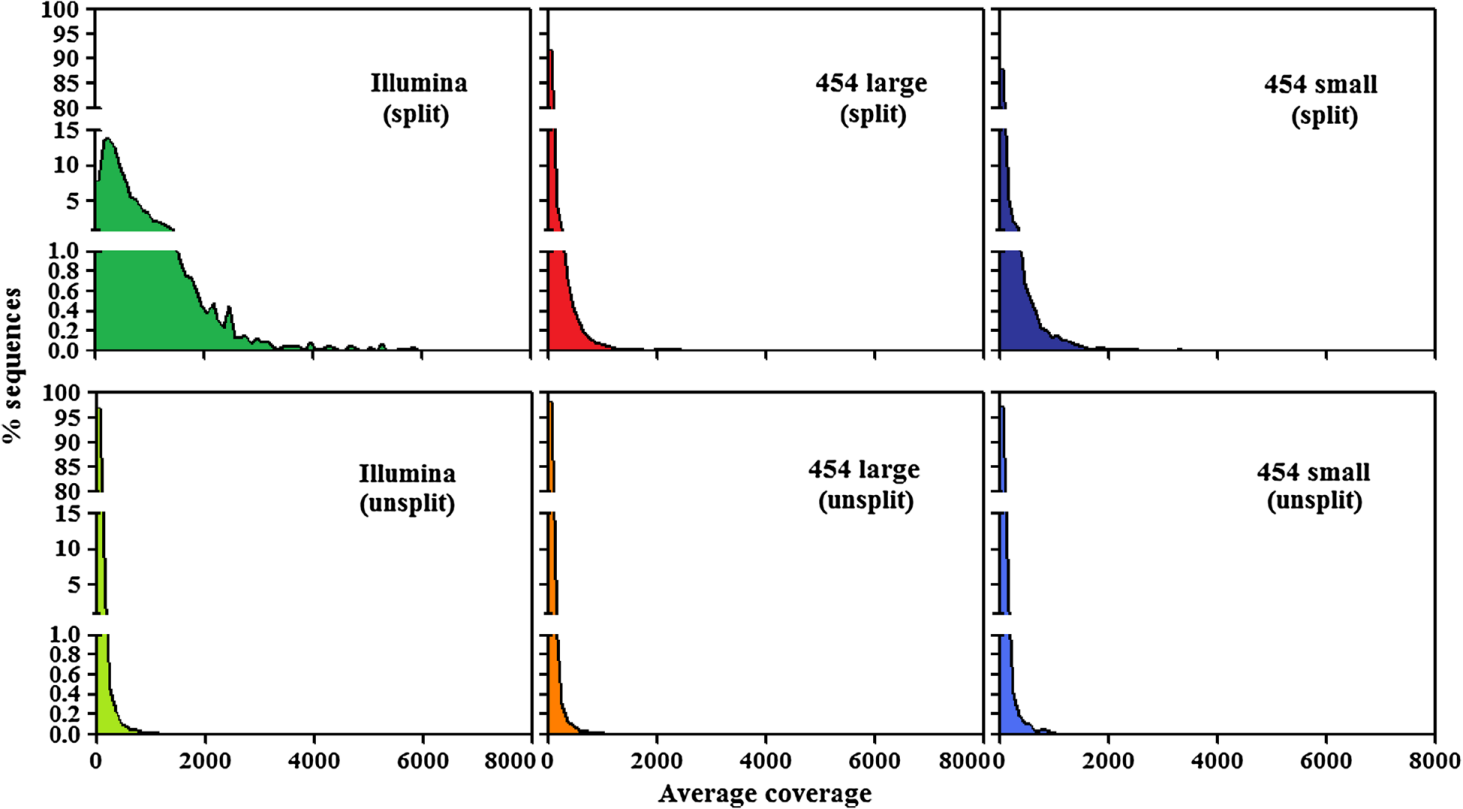
Distributions of mapped Illumina reads to the six sequence sets obtained by assembling original Illumina or 454 reads.

By varying sequencing technology (Illumina or 454), coverage (0.55 ×-1.25 ×), assemblers and assembly procedures (with or without splitting of read packages), different genomic databases were produced. On the whole, it can be observed that for each of three packages of reads (Illumina, 454 large, and 454 small read packages) the split subpackages resulted in the production of a lower number of contigs (Table 1). However, contigs were far more repetitive than those produced by simple assembly of whole reads, as shown by higher values of average coverage (Table 1, Figure 1). In fact, sequences assembled from the split sets were from about three-fold (for 454 large package) to more than 27-fold (for Illumina reads) more repeated in the genome than those assembled from unsplit sets.

**Table 1:** Characteristics of contig sets obtained by CLC Bio Workbench and Minimus2 assemblies after different splitting of Illumina and 454 reads

**Sequence read types**	**Nr. of sub-packages**	**Subpackage coverage**	**Nr. of assembled contigs**	**Mean length**	**Mean average coverage**	**N**_**50**_
Illumina	1	0.86 x	28,283	505.4	22.1	487
	565	0.0015 x	4,599	542.9	606.1	519
Total			32,377	513.4	205.8	496
454 (large)	1	1.25 x	144,755	627.7	13.8	678
	26	0.048 x	133,900	595.3	39.2	610
Total			227,160	662	42.6	718
454 (small)	1	0.55 x	42,964	457.6	16.6	449
	18	0.031 x	28,984	466.1	57.5	455
Total			59,923	484.7	106.1	478

**Figure 2:**
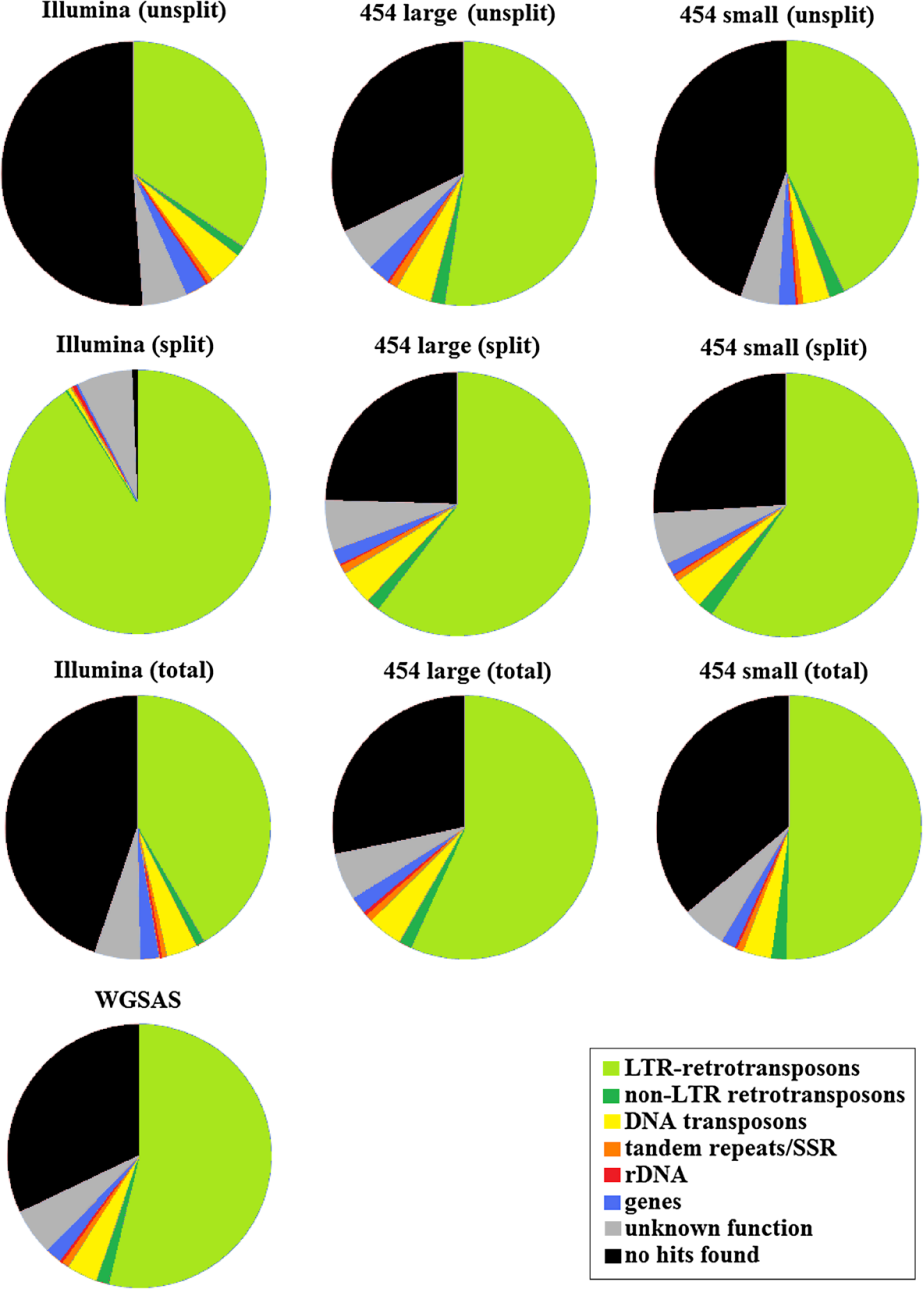
**Functional composition of the assembled sequence sets.** obtained by assembling original Illumina or 454 read sets (first row, unsplit), by assembling the same read sets after a preliminary splitting into subpackages of reads (second row, split), by assembling the two assembled sequence sets previously obtained from Illumina, 454 large and 454 small sets of reads (third row, total), and by assembling the three assembled sequence sets described in the third row (fourth row, WGSAS).

The annotations of the six sets of assembled sequences show large differences in functional composition (Figure 2). Differences were especially pronounced when the same set of reads was split into subpackages prior to assembly. Figure 2 shows that low redundancy sequences such as putative genes or non-LTR retrotransposons were more common when the assemblies were conducted with no preliminary splitting. In contrast, preliminary splitting resulted in the assembly of larger percentages of LTR-retrotransposons. This is especially true for Illumina reads (Figure 2), probably because of their shorter length compared to 454 reads. Also, contigs for which no significant similarity was found in the existing databases were more (and in certain cases, much more) frequent in the unsplit assembled read set (Figure 2).

The six assembled sequence sets (with and without splitting) from the Illumina, 454 large, and 454 small sets of reads were each assembled two by two (split and unsplit) and annotated. The functional composition of the three resulting assemblies was similar, except that the frequency of retrotransposons was higher in both 454 packages than in the Illumina read set. In addition, a larger frequency of unclassified sequences was obtained using the Illumina read set (Figure 2).

Because of the large differences in average coverage and functional composition among the six assembled sequence sets, a further assembly was performed to produce a comprehensive genomic sequence set for sunflower. A total of 283,800 sequences (including 54,427 supercontigs and 229,538 individual contigs) were obtained, representing a whole genome set of assembled sequences (WGSAS).

The reliability of this method to obtain accurate sequences was tested by comparison of these sequences to available, Sanger-sequenced ones. Twenty alignments between assembled contigs and real sunflower DNA sequences are shown as Additional file 1. Mismatches related to transitions/transversions represent only 7.1% of 12,727 aligned nucleotides, indels amount to 0.4%.

### SUNREP, a database of sunflower repetitive sequences

**Figure 3:**
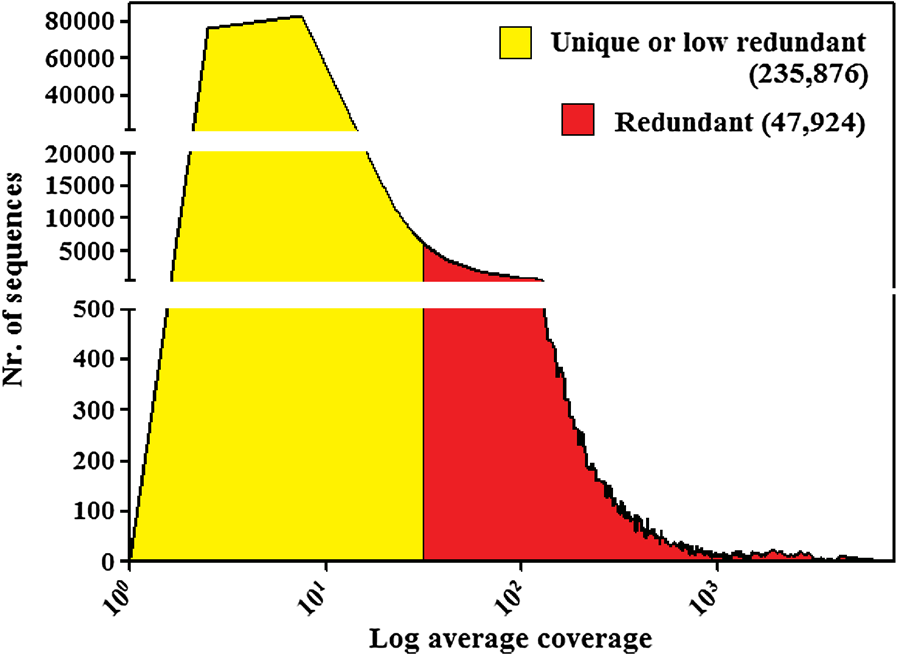
**Distribution of mapped Illumina reads in the WGSAS.** Sequences were subdivided into redundant and unique (low redundant), based on an arbitrary value corresponding to five-fold the mean average coverage of five putatively unique gene sequences.

The WGSAS was mapped with the large set of Illumina reads as above. The distribution of average coverage of the WGSAS is reported in Figure 3. The average coverage was used as a parameter by which the repetitive sequences could be discriminated from the others. In plants much of the genome may be repeated because of the polyploidy events that have occurred during their evolutionary history ([35], as an example). Therefore, we evaluated sequence redundancy in relation to the average coverage of five sunflower gene sequences that were considered as unique reference sequences. By mapping Illumina reads to the WGSAS to which the five genes were added, we obtained for those sequences an average coverage of 6.6. We conservatively identified as repeated sequences all of those contigs with an average coverage higher than five-fold the mean average coverage of the five reference sequences (6.6 × 5 = 33.0). By this method, we identified 47,924 repeated sequences that constitute a database of repetitive sequences of sunflower, hereafter called SUNREP. The remaining 235,876 sequences of WGSAS were classified as unique or low redundant.

The distribution of different sequence types in SUNREP is reported in Table 2. It can be observed that 11.50% of sequences included in SUNREP did not find any hits in the public databases used for annotation. Among the annotated sequence types, retrotransposons were by far the most represented in SUNREP. Of LTR-retrotransposons, sequences belonging to the *Gypsy* superfamily were 2.3-fold more represented than those belonging to the *Copia* superfamilies.

**Table 2:** Functional distribution of the sequences in the SUNREP database

**Sequence type**		**Number**
DNA transposons	Unclassified	373
	Tc1 Mariner	5
	hAT	67
	Mutator	101
	PIF-Harbinger	18
	CACTA	64
	Helitron	324
	MITE	382
Retrotransposons	Unclassified	192
	LTR-*Copia*	8,605
	LTR-*Gypsy*	19,726
	LTR-Unknown	5,636
	Non-LTR	261
	Pararetrovirus	11
Tandem repeats and SSR		385
rDNA		84
Putative genes		483
Unknown repeats	Unclassified	4,739
	Contig 61 type [18]	957
No hits found		5,511
Total		47,924

Interestingly, a large fraction of sequences showed similarity to LTR-retrotransposons, but the superfamily could not be determined. Such elements lack coding sequence, are non-autonomous and usually species-specific. They can be discovered only when long sequences are available because their identities are based on structural features and not on sequence similarity to retrotransposon coding domains. In this study, we identified these elements only by their sequence similarity to those first reported by Buti et al. [22]. Non-LTR retrotransposons were poorly represented, as frequently observed in plant genomes.

Putative DNA transposons accounted for 1,334 sequences. A portion of these were classified as DNA transposons according to sequence similarity to the short domain of the transposase gene. All types of plant DNA transposons were putatively found in SUNREP, with a prevalence of MITEs and Helitrons.

**Figure 4:**
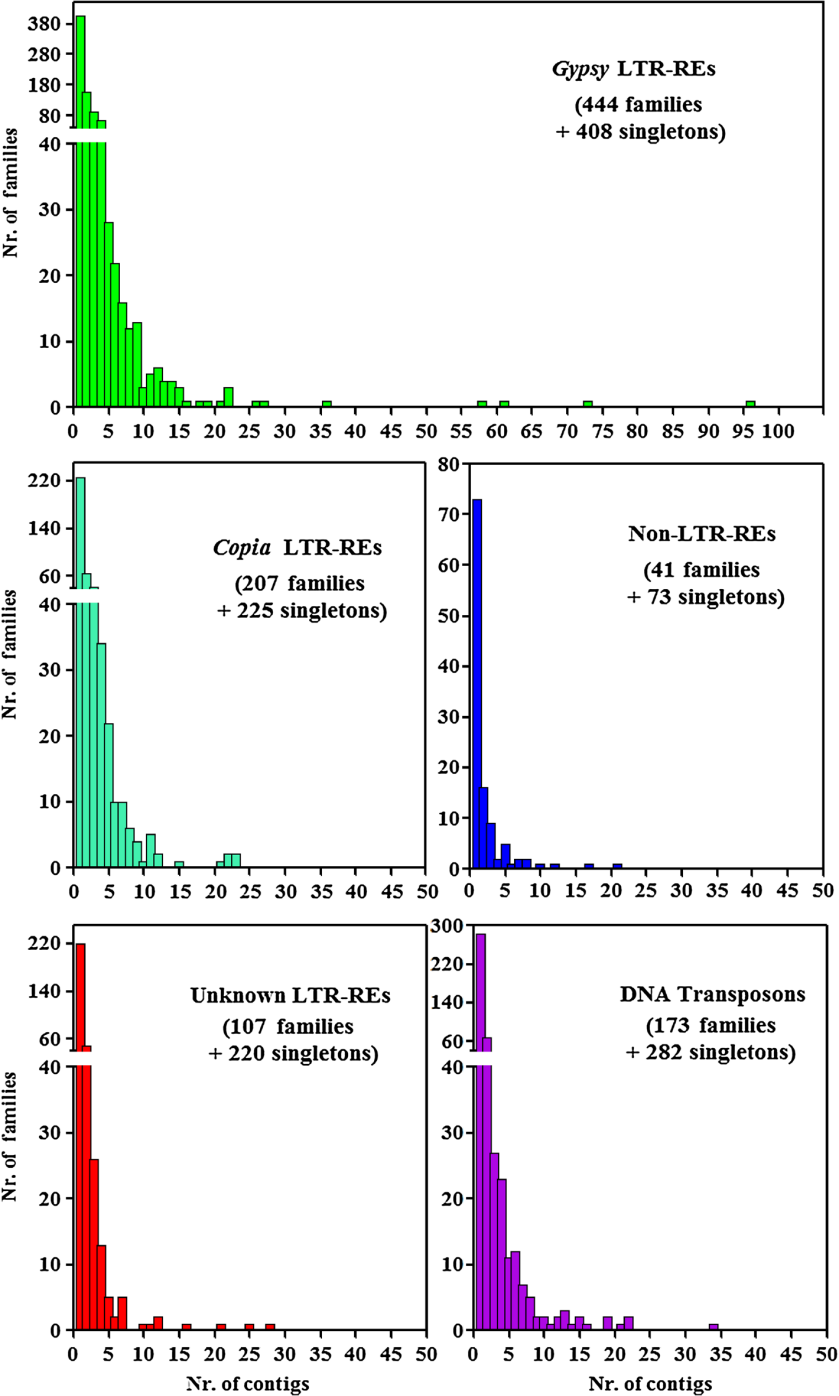
**Size distribution of *Gypsy, Copia, *and unknown LTR REs, of non-LTR REs, and of DNA transposons families obtained performing an all-by-all BLAST analysis.** For each superfamily, the histograms depict the number of families (Y-axis) containing a specified number of contigs. The total number of families and singletons (i.e. families represented by one contig) are also reported.

SUNREP contigs showing sequence similarity to LTR-REs, non-LTR REs, and DNA transposons were also analysed using an all-by-all BLAST search to estimate the occurrence in SUNREP of similar sequences within those repeat classes, i.e. sequences that were assembled separately, even though sharing some sequence similarity. Each class of repeats was subdivided into families (i.e., composed by at least 2 SUNREP sequences) and singletons (i.e., sequences that did not share similarity). The distribution of such families according to the number of sequences for each of them is reported in Figure 4.

**Figure 5:**
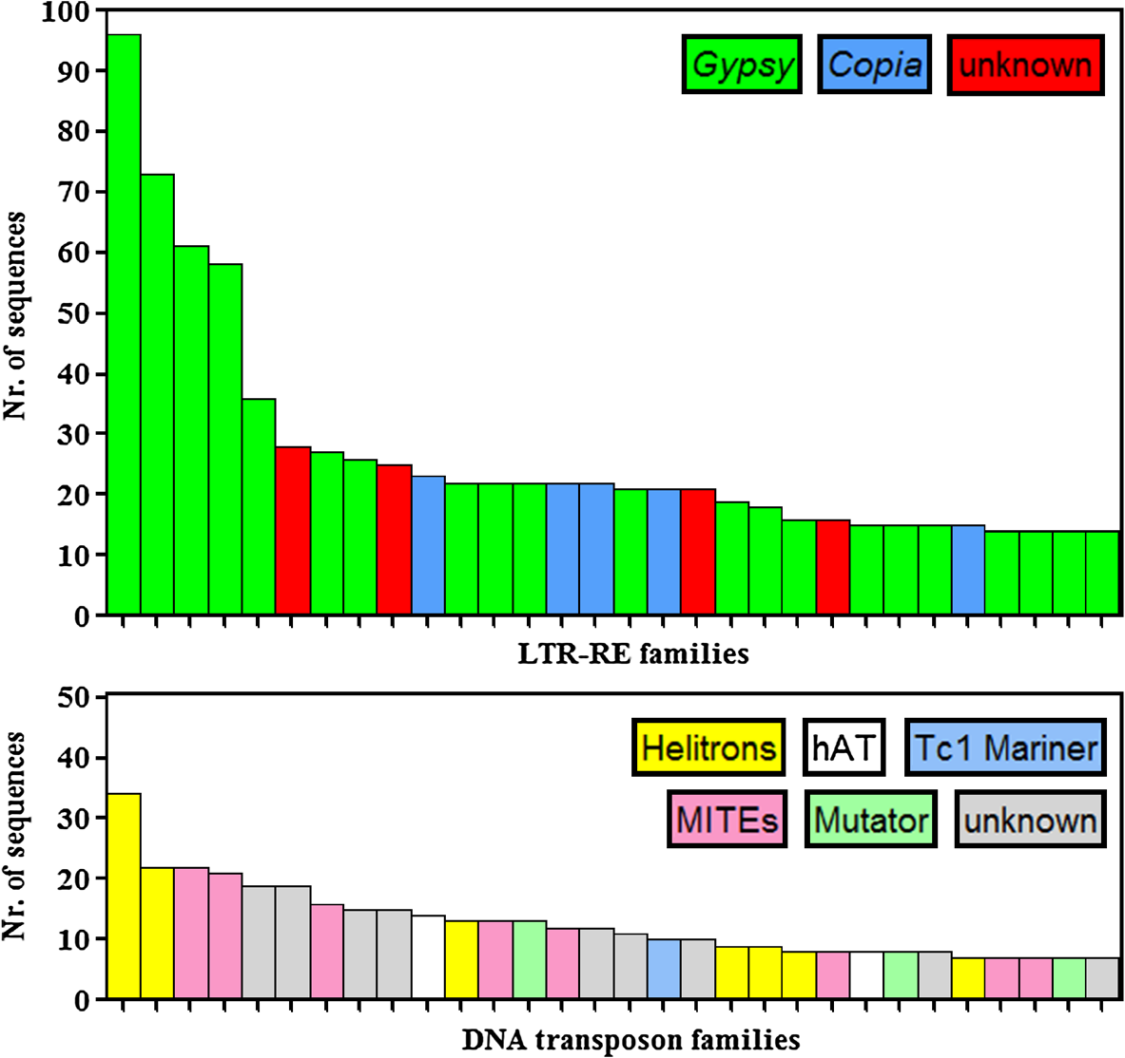
Number of sequences composing the 30 most numerous families of LTR-REs (above) and DNA transposons (below).

The most redundant family, belonging to the *Gypsy* repeat superfamily, included only 96 of the 47,924 sequences of SUNREP (0.20%). Only four *Gypsy* families were composed of more than 50 SUNREP sequences. Considering the 30 most numerous LTR-REs families, the vast majority belonged to the *Gypsy* superfamily (Figure 5). Among the 30 most numerous DNA transposons, the most common families belonged to the Helitron class, followed by putative MITEs (Figure 5). It should be noted that the number of sequences that belong to a family in SUNREP does not reflect the redundancy of that family in the genome.

Another class of repeats was classified as unknown, but showed similarity to sequences previously isolated and whose redundancy has already been measured in sunflower by molecular and bioinformatic procedures [20]. These unknown repeats were largely represented in SUNREP. Many of these showed similarity to the most repeated sequence identified by Cavallini et al. [20], the so-called Contig 61, whose nature remains to be ascertained.

Finally, according to BLAST analysis, 483 sequences of SUNREP showed similarity to putative protein encoding sequences. Of these, 123 were classified as hypothetical proteins, without any further annotation, the others belonged to 199 different gene families, of which 5 were represented by at least 9 sequences in the database (Table 3).

**Table 3:** The most abundant gene families represented in the SUNREP database

**Protein encoded by the gene family**	**Nr. of sequences**
NBS-LRR Disease Resistance Protein	24
DNAJ-like Protein	18
Protein Kinase Domain Containing Protein	13
F-box Motif Containing Protein	10
Serine/Threonine/Tyrosine Protein Kinase	9

The most redundant genes encode the NBS-LRR class of proteins, receptors that recognize highly variable pathogen effectors [36]. Another redundant gene family encode DNAJ proteins. These proteins function in association with Hsp70 molecular chaperones to facilitate protein folding and play an active role in regulating normal cellular events like protein degradation, morphogenesis and cell cycle progression [37].

The third redundant gene family is very heterogeneous, encoding proteins with un-specified protein-kinase domains, that are involved in the transduction of signals to binding factors, to the centromeres, and to other effectors. Beside aspecific kinases, also serine/threonine/tyrosine kinases are encoded by a redundant gene family. The last redundant and heterogeneous gene family encodes F-box motif containing proteins.

### Sunflower genome composition

In the case of a small insert library [20] or of a whole genomic shotgun sequence library [21], the composition of the sequence set directly reflects the composition of the sunflower genome. Conversely, in the case of a sequence set obtained by assembling Illumina and 454 reads, the simple composition of the set cannot offer a picture of the genome composition, because repeated sequences are assembled together and hence are underestimated. Consequently, we evaluated the composition of the sunflower genome by counting the number and percentage of reads that mapped to each sequence in the WGSAS. Mapping results are summarized in Table 4.

**Table 4:** Statistics of the mapping of Illumina reads to the WGSAS

	**Sequence type**	**Number of reads**	**% of total nuclear reads**	**% of matched nuclear reads**
Matched nuclear reads	Repeated	61,860,742	52.13	80.92
	Unique or low redundant	14,586,474	12.29	19.08
	Total	76,447,216	64.42	100.00
Not matched nuclear reads		42,217,465	35.58	
Total nuclear reads		118,664,681	100.00	
Organellar reads		11,680,927		
Total reads		130,345,608		

Based on their similarity to the sequences in the organellar database, we estimated that more than 11.6 millions of reads were of organellar DNA origin. Regarding other reads, around 42 million reads did not match any assembled sequence, indicating that the WGSAS does not cover the entire genome, as expected having assembled only a total of 2.66 × coverage. It is likely that much of the missing sequences were low copy-number regions in the genome and that the relatively low coverage used in our study did not allow assembly of such loci. Such low-copy sequences could be protein encoding genes or rare forms of repeats whose sequence was degenerated until becoming unique. Some of these unmapped reads also likely represent sequencing errors of some kind. On the other hand, it is also possible that stringent assembly procedures and shorter reads affecting alignment stringency have contributed to increase the number of unaligned reads.

Considering that Illumina reads in our experiments were sampled without bias for particular sequence types, the percentage of reads that matched to a sequence class indicated the proportion of that sequence class in the sunflower genome. So, it was estimated that the percentage of repetitive sequences in the *H. annuus* genome was very high, amounting at least to 52.13% (see Table 4), while unique or low redundant sequences (that should include the vast majority of protein-encoding genes) represented only 12.29% of the genome at least. The rest (35.58%) of the genomic reads did not match any contigs.

Sunflower genome composition was estimated also in terms of sequence types. The frequency of each repeat type was calculated based on mapping the WGSAS with the 4 x coverage of Illumina reads and counting the number of reads matching each sequence type. Such frequencies are reported in Table 5, adopting the nomenclature proposed by Wicker et al. [24].

**Table 5:** Percentage distribution of different functional classes of non-coding DNA sequences in the sunflower genome, based on the mapping of the WGSAS

**Sequence type**		**Whole genome sequence set**	
		**Number of matched reads**	**Percentage**
DNA transposons	Unclassified	521,152	0.68
	Subclass I	445,239	0.58
	Subclass II	348,166	0.46
	MITE	641,043	0.84
	Total	1,955,600	2.56
Retrotransposons	Unclassified	347,042	0.45
	LTR-*Copia*	14,693,697	19.22
	LTR-*Gypsy*	37,625,059	49.22
	LTR-Unknown	7,569,830	9.90
	Non-LTR	541,494	0.71
	Pararetrovirus	20,624	0.03
	Total	60,797,746	79.53
Tandem repeats		457,613	0.60
rDNA		266,528	0.35
Unknown repeats	Unclassified	3,888,190	5.09
	Contig 61 type [18]	2,148,599	2.81
	Total	6,036,789	7.90
Total matched reads excluding organellar ones		76,447,216	

It can be observed that retrotransposons (especially LTR-retrotransposons) were by far the most abundant class of sequences in the sunflower genome, accounting for at least 79.53% of the reads matching the WGSAS, while DNA transposons and non-LTR retrotransposons showed very low percentages (Table 5). Of LTR-retrotransposons, the vast majority belonged to the *Gypsy* superfamily, which is 2.56-fold represented compared to *Copia* superfamily. A large amount of the genome (9.90%) was apparently made up of LTR-retrotransposons of unknown superfamily. It is presumable that their frequency in the sunflower genome was underestimated, and will increase after the sunflower genome sequence becomes available.

In other analyses we mapped Illumina reads to a sample of 19 intact LTR-retrotransposons of sunflower, isolated by Buti et al. [22], to estimate the equilibrium between retrotransposon replication and retrotransposon loss. Illumina reads were mapped to these retrotransposons, keeping separated LTR sequences from the respective inter-LTR region (that is the encoding region for *Gypsy* and *Copia* retroelements and an apparently non-encoding sequence for unknown retroelements, respectively). The results of mapping are reported in Table 6. It can be observed that the ratios between LTR and inter-LTR average coverage ranged from 0.01 to 6.58. If all retrotransposons belonging to one and the same family were intact, i.e. composed by two LTRs and one inter-LTR region, the ratio should have been 2. For 9 out of 19 analysed LTR-REs the ratio was higher than 2, indicating the occurrence of solo-LTRs of that RE family in the genome. The other LTR-REs had a ratio ranging from 0.01 to 1.25, i.e. the inter-LTR region was more represented in the genome than the LTR. This result can be explained by the presence of different families that share, at least in part, the inter-LTR region. Interestingly, analysing separately *Gypsy*, *Copia*, and unknown elements, the mean ratio between LTR and inter-LTRs average coverage was higher than 2 only for *Gypsy* elements (Table 6).

**Table 6:** Average coverage of a sample of full-length sunflower LTR-retrotransposons measured separately on LTR and inter-LTR regions

**Superfamily**	**Sequence code **[22]	**Average coverage**		**LTR to Inter-LTR ratio**
		**LTR**	**Inter-LTR**	
*Copia*	DESRLC1	1037.85	830.65	1.25
	DHNRLC1	186.63	3303.45	0.06
	LTPRLC1	62.72	13.47	4.66
	LTPRLC2	12.54	1367.95	0.01
	LTPRLC3	340.37	415.11	0.82
	mean			1.36
*Gypsy*	DESRLG1f	9239.29	2288.53	4.04
	DESRLG2	2337.58	659.22	3.55
	DESRLG3	14003.10	3932.65	3.56
	DHNRLG1	2345.90	560.36	4.19
	DHNRLG2	976.16	1776.89	0.55
	LTPRLG1	6267.76	10258.53	0.61
	LTPRLG2	823.73	125.25	6.58
	LTPRLG3	6016.77	1024.56	5.87
	mean			3.62
Unknown	DESRLX1	234.20	104.46	2.24
	DESRLX2	2064.22	1827.43	1.13
	DHNRLX1	702.73	5577.56	0.13
	DHNRLX2	519.09	791.34	0.66
	LTPRLX1	1053.57	1875.62	0.56
	LTPRLX2	956.69	356.17	2.69
	mean			1.23
Mean				2.27

## Discussion and conclusion

In our experiments, different strategies were used for assembling original sunflower sequence reads and for obtaining contigs; i.e. different packages of reads (Illumina and 454) were subdivided into low-coverage subpackages prior to assembly.

Similar levels of sequence coverage have proven to be efficient in generating a considerable amount of biologically useful information and genomic resources in other species [15,38]. By using low genome coverage, most of the assembled contigs do not represent specific genomic loci; instead, they are probably composed of reads derived from multiple copies of repetitive elements, thus representing “consensus” sequences of genomic repeats [39]. Even though the exact sequence of this consensus does not necessarily occur in the genome, this representation of repetitive elements is sufficiently accurate to enable amplification of whole length repetitive elements by PCR [38]. Indeed, our comparison of assembled contigs with available Sanger sequences demonstrates good correspondence between virtual and real sequences.

Our results clearly show that splitting the original packages of reads into a number of subpackages allowed us to assemble more contigs similar to repetitive sequences, although assembled contigs were fewer (in the case of Illumina reads, much fewer) than those obtained by assembling the sets of reads prior to splitting. The difference in number and redundancy of assembled sequences was more striking for Illumina reads than for 454 reads, probably due to the short length of Illumina reads. However, splitting the packages of reads did not apparently affect the mean length and the N_50_ of the assembled contigs.

The sequence sets obtained by using different pre-assembly approaches were different in sequence types and redundancy. Hence, the production of a WGSAS by further assembling of the different sets provided a more complete picture of sunflower genome composition. In addition, the analysis of redundancy based on mapping Illumina reads onto the WGSAS allowed us to quantify the redundancy of each contig.

Sunflower genome composition has already been ascertained using other methods, i.e. biochemical analyses [40], sequencing and analysis of a small insert library [20], whole genome 454 sequencing [21]. All these analyses may have some potential weakness. Biochemical analyses [40] obviously do not consider DNA sequence but only denaturation and reassociation kinetics of DNA, so rare forms of repeats are excluded such as, for example, retrotransposon remnants. The Sanger-sequenced small insert library [20] comprised only 1,638 sequences, so conclusions are subject to sampling errors. The whole genome 454 sequence database [21] is based only on sequence similarity, however the number of 454 reads used (total coverage 0.23 ×) might be not sufficient to ensure accurate estimation of genome composition.

The present analyses showed that sunflower genome is mostly composed of LTR-retrotransposons (78.8%), similar to that already reported [21,34]. It is known that the genome size is determined during evolution by an equilibrium between enlargement of the genome by insertion of REs and RE-mediated DNA removal [41-43]. DNA rearrangements, illegitimate recombination, and unequal homologous recombination drive DNA removal in plants by a number of mechanisms, such as the repair of double strand breaks (nonhomologous end-joining) and slipstrand mispairing [44-49].

The observed large number of retrotransposons indicates that such elements have been actively replicating during the evolution of this species. Recent studies have reported that sunflower LTR-REs are transcribed even at present [20,26-28] and, in at least one case, RE transcription was shown to be followed by RE insertion [27].

Mapping Illumina reads to a set of 19 available intact LTR-retrotransposons suggested the occurrence of numerous solo-LTRs for 9 out of 19 REs, although the occurrence of REs sharing LTRs but having different internal regions cannot be ruled out and could lead to an overestimation of solo-LTR frequencies. Solo-LTRs are typically produced by illegitimate recombination. Our data suggest that massive amplification of these elements in the sunflower genome was partly counterbalanced by substantial DNA loss, especially related to *Gypsy* elements, although in other studies solo-LTRs have been found commonly for *Copia* elements as well [20,21]. It is obvious that a very large number of intact retroelements are needed to validate this analysis.

Concerning the different RE superfamilies, the ratio between *Gypsy* and *Copia* retrotransposon frequencies amounted to 2.29, confirming the greater abundance of the former superfamily. This ratio is generally species-specific. *Gypsy* to *Copia* frequency ratio is even higher in papaya (5:1, [50]), *Sorghum* (4:1, [51]), and rice (3:1, [52]) than in the sunflower genome. In other cases, as in maize [53], poplar [54], and olive (Barghini, personal communication) a similar abundance of the two superfamilies was observed. Finally, in grapevine an opposite trend was found, with *Copia* elements two-fold more represented than *Gypsy* ones [35].

The large abundance of *Gypsy* elements compared to *Copia* can be explained by two hypotheses: *Gypsy* elements have been more active during sunflower evolution and/or they have been active more recently, so that are more easily recognizable by similarity searches, having been subjected to fewer mutations. Dating retrotransposon insertions in the sunflower genome indicate that *Gypsy* elements are generally younger than *Copia*, though some *Copia* elements are relatively young as well [21,22].

Retrotransposon and DNA transposon sequences included in the redundant fraction of the WGSAS (SUNREP) were also assigned to different families within each superfamily, by an all-by-all BLAST search. The number of sequences composing each family was generally low, confirming that there are not prominent transposon families in this species [20,21].

In a previous study [21], a different approach was used for determining the composition of different repeat types in terms of families, by using the graph-based method of Novak et al. [39]. The families of LTR-retrotransposons and DNA transposons generally match the results reported in Staton et al. [21], with the exception of putative MITEs, that are more frequent than previously observed in other studies. Interestingly, the most frequent DNA transposon family belongs to the Helitron superfamily and is comprised of a number of sequences comparable to that of the most numerous LTR-RE subfamilies. Also the graph-based study included one Helitron subfamily among the 20 most redundant ones in the sunflower genome; all the others belonging to the LTR-RE class.

The results obtained by Staton et al. [21] and those reported in this study indicate that both the method by Novak et al. [39] and the all-by-all BLAST search (performed in our experiments) allow a precise estimation of repeat superfamilies and families. The first method allows information to be gained on repeat structure and provides putative consensus sequences of the repeat; all-by-all BLAST search (preceded by assembling all available sequences) can be applied to larger sets of reads.

Finally, mapping data indicated that a number of contigs showing similarity to putative protein encoding genes are to be considered as redundant. In many cases such contigs showed similarity to gene families already known to be repeated in plant genomes, such as NBS-LRR genes [36]. In other cases, it is likely that functional domains, and not genes, are the cause of apparent redundancy. For example, F-box proteins are identified by the presence of protein interaction domains that bind ubiquitinilation targets and include a large variety of proteins [55]. For other contigs, it might be that a gene (or a gene fragment) lies close to a repeated sequence and the redundancy of that contig is related to the repeated sequence and not to the gene sequence. Interestingly, our data indicated the occurrence, in the sunflower genome, of a relatively high number of putative Helitrons. These sequences, of transposable origin, are known to include DNA retro-transcribed on RNA transcripts [5] and might be responsible for the relatively high frequency of gene fragments in the redundant fraction of the WGSAS.

In conclusion, the results of our experiments show how different data partitioning and assembly approaches can be used to obtain valuable insights on genome composition using NGS technologies, either 454 or Illumina, or both technologies combined. Concerning sunflower, our data confirm the repeat structure of the genome and give new insights on different aspects of it. Moreover, they will facilitate the functional annotation of the *H. annuus* genome that is currently being sequenced and will be used for studies of intra- and interspecific variability related to *H. annuus* and its relatives.

## Methods

### Whole-genome-shotgun sequencing

Leaf tissue was sampled from a single individual from a highly inbred sunflower cultivar HA412-HO (PI 642777). Total genomic DNA was extracted using a CTAB procedure [56] and randomly sheared into fragments for sequencing.

For Illumina sequencing, library preparation followed the standard multi-step Illumina protocol [57]. Ligated, size-selected fragments (~280 - 320 bp) were amplified through 18 cycles of polymerase chain reaction (PCR), using Phusion High-Fidelity PCR Master Mix (New England Biolabs, Ipswich, MA, USA) and standard Illumina primers. Resulting product concentrations were determined using a Nanodrop 1000 (ThermoFisher Scientific, Wilmington, DE, USA).

Enriched, cleaned product was diluted to 5 pM and submitted for sequencing on an Illumina GAII sequencer at the University of British Columbia (Vancouver). Denaturation, cluster generation, and subsequent sequencing followed the manufacturer’s recommendations. Image analysis, base-calling and error estimation were performed using the Illumina GA Pipeline version 1.5. Low quality bases, empty reads, and adapter sequences were removed using CLC-BIO Genomic Workbench, version 5.1 (CLC-BIO). A total of 28,236,626 random genomic sequences (mean read length 101 nt, total coverage 0.86 ×) were obtained. In a second experiment, 130,345,608 Illumina reads (mean read length 101 nt, total coverage 4 ×) were obtained.

In other experiments, standard 454 sequencing runs were performed on a Roche 454 GS FLX sequencer (Roche, http://www.roche.com) with XLR (Titanium) chemistry at The McGill University and Génome Québec Innovation Centre (Montreal, Canada). We obtained 22,666,169 WGS sequences, for a total of 1.8 × coverage, subdivided in two packages, a large one (mean read length 166.2 nt, coverage 1.25 ×), and a small one (mean read length 349.4 nt, coverage 0.55 ×).

### Assembly procedures

In a first approach, an Illumina read set (genome coverage 0.86 ×), 454 large read set (1.25 ×), and 454 small read set (0.55 ×) were assembled separately. Each read set was assembled using CLC-BIO based on unambiguous overlaps. The resulting contigs were further assembled separately using Minimus2 software [58]. CLC-BIO assembly parameters were: minimum contig length = 300; minimum distance = 200; max distance = 600 for Illumina package; minimum contig length = 300 for 454 large package; and minimum contig length = 300; minimum distance = 5,000; max distance = 15,000 for 454 small package. Minimus2 assembly parameters were REFCOUNT = 0 and MINID = 90. This second assembly produced three sets of supercontigs and single contigs. The resulting contigs were assessed as to their number, length, and N_50_ (Table 1).

In a second approach, each of the three read sets was split into low coverage subpackages. For the 454 large and small sets of reads, the split was performed to obtain 26 and 18 subpackages, with 0.048 and 0.031 × coverage, respectively. For the Illumina reads, we prepared 565 subpackages, each with less coverage (0.0015 ×) than those used for 454 reads, because preliminary experiments showed us that this level of coverage allows the largest recovery of repeated sequences (Barghini, personal communication). Each subpackage was individually assembled using CLC-BIO, then each group of subpackages was further assembled using Minimus2 with the above described parameters. This second assembly produced three additional sets of supercontigs and single contigs. Possible contaminants resembling organellar sequences were then removed from the six sets of contigs by masking them against an in-house sunflower organellar sequence database using RepeatMasker (http://www.repeatmasker.org/), and excluding all contigs showing at least 1% of their length similar to organellar sequences. The remaining contigs of the six sets were further assembled, using Minimus 2 with REFCOUNT = 0 and MINID = 90: in a first step, the six sets of contigs were assembled two by two (split and unsplit), to produce three sets of sequences; in a second step, the three sets were assembled into a unique set of assembled sequences. For all assemblies also, the resulting contigs were assessed for number, length, and N_50_ (Table 1).

### Redundancy estimation of sequences

Relative redundancy of each sequence in the six sets of assembled sequences and in the WGSAS was estimated by mapping the sequences with a large Illumina sequence read set (total coverage 4 ×). Mapping was performed using CLC-BIO, which randomly places multi-reads, hence the number of mapped reads to a single sequence is only an indication of its redundancy. On the other hand, if all sequences of a repeat family or class are taken together, the total number of mapped reads (in respect to total genomic reads) indicates the effective redundancy of that family or class. To establish mapping parameters, sixty sequences were selected for which redundancy had been previously determined by slot blot and hybridization ([20]; Giordani, personal communication). For these sequences, correlations were calculated between their known redundancy and their average coverage (the sum of the bases of the aligned part of all the reads divided by the length of the reference sequence) by using different parameters (mismatch cost, deletion cost, insertion cost, length fraction, similarity, Additional file 2). The parameters determining the largest correlation were selected to be used in the subsequent mapping of different sequence sets. The means and distributions of average coverage values for each contig of the six sets are reported in Table 1 and Figure 1, respectively.

In the case of the WGSAS, to evaluate the redundancy of DNA sequences, the same 4 × Illumina sequence read set was mapped onto the WGSAS plus one actin-encoding gene (FJ487620.1) and four unique gene sequences [59], encoding a lipid transfer protein (FR671365.1), a z-carotene desaturase (FR671183.1), an auxin-binding protein (FR671175.1), and an ABA-responsive C5 protein (FR671167.1). Then the average coverage was calculated for each gene sequence. We established an average coverage of 33.0 (i.e., five-fold that of the mean average coverage of the five above sequences, 6.6) as an arbitrary threshold for discriminating repeated sequences from unique or low-redundant ones.

The mapping procedure, using CLC-BIO and the same 4x Illumina sequence read set as above, was adopted for determining the relative redundancy of LTR and inter-LTR regions of a sample of sunflower retrotransposons.

### Annotation of repetitive sequences

Sequences belonging to all assembled sequence sets were searched for homologies by using the NCBI BLAST with an E-value cut-off of 10^-10^ in the NR NCBI database (http://blast.ncbi.nlm.nih.gov/), containing all the non-redundant protein sequences, in the database RepBase [60] and in other available sunflower sequence sets [20,22]. One region might match two or more homologous elements in the database. We then removed the redundant annotations by keeping only the region with the longest match and significant E-value. We also performed an analysis of SUNREP by using RepeatMasker to isolate microsatellites and low complexity sequences.

All sequences belonging to the WGSAS that were not included in SUNREP were annotated using RepeatMasker against SUNREP sequences, to isolate and annotate low copy remnants of repeated sequences, as transposons that had accumulated mutations.

After annotation, all SUNREP sequences similar to LTR-REs, non-LTR-REs, and DNA transposons were grouped into families according to their sequence similarity by performing an all-by-all search using BLAST with the following parameters: -r 4 -q −5 -e 1e-50. For each sequence, the most similar sequence (with a similarity score E^-50^ at least) in the database was recorded. Then, each sequence sharing similarity was attributed to the same family.

## Competing interests

The authors declare that they have no competing interests.

## Authors’ contribution

Conceived and designed the study: LN AC; Generated the sequence data: NG NCK LR; Performed assembling, redundancy analyses and annotation of sequence data: RMC; Participated in performing assembling, redundancy analyses and annotation of sequence data: EB TG MB FM; Participated in the interpretation and discussion of results and contributed to the writing of the paper: LN RMC EB TG MB FM MM NG NCK LR AC; Principal investigator for the “SUNREP” PRIN-MIUR project and coordinator of the study: LN; Wrote the paper: LN, TG, NCK, LR, and AC; All authors read and approved the final manuscript.

## Supplementary Material

Additional file 1 **Alignment of a sample of 20 assembled sequences of the sunflower whole genome database (inbred line HA412-HO, numerical codes, below) to Sanger sequences from the small insert library **[20]. Click here for file

Additional file 2 **The 5 sets of CLC-BIO parameters used for mapping of the Illumina reads to 60 sunflower DNA sequences with known redundancy **([20]; **Giordani, personal communication) and correlation between known copy number and average coverage.** Click here for file
