# Supplementary material for: The repetitive component of the sunflower genome as shown by different procedures for assembling next generation sequencing reads
Source: BMC Genomics. 2013 Oct 6;14:686. doi: 10.1186/1471-2164-14-686 (PMC3852528; doi:10.1186/1471-2164-14-686)
Supplement: Additional file 1 — Alignment of a sample of 20 assembled sequences of the sunflower whole genome database (inbred line HA412-HO, numerical codes, below) to Sanger sequences from the small insert library [20]. [file 1471-2164-14-686-S1.pdf]

Additional file 1. Alignment of a sample of 20 assembled sequences of the sunflower whole genome database (inbred line HA412-HO, numerical codes, below) to Sanger sequences from the small insert library described in Cavallini et al. (2010) (inbred line HCM, HAG codes, above)

```
Query= HAG001C01F
Subject= 73340

HAG001C01F: 1 ccgtctagacaagttgtgtttcatcaagaggcatcagttaagacctgttcatataaatat 60
              |||||||||||||||||| |||||||||||||| ||||||||||||||||||||||||||
73340:    982 ccgtctagacaagttgtggttcatcaagaggcaccagttaagacctgttcatataaatat 923


HAG001C01F: 61 tttgtctcctgcaagcccagagactttacaggagaaaatggggccatcgattgcatgaca 120
               ||| ||||||||||||||||||||||||||||||||||||||||||||||||||||||||
73340:     922 tttatctcctgcaagcccagagactttacaggagaaaatggggccatcgattgcatgaca 863


HAG001C01F: 121 tggcttgatgaaatggacgctgttgttgacatcagcgattgtgcggagcaagatgttgtg 180
                ||||| ||||| ||||||||||||||||||||||||| ||||||||||||||||||||||
73340:      862 tggctcgatgagatggacgctgttgttgacatcagcggttgtgcggagcaagatgttgtg 803


HAG001C01F: 181 aaatttgtttcacaatcatttaaaggagaagcgttggcatggtggaggtcattactccaa 240
                ||||||||||||||||||||||||||||||||||||||||||||||| ||||| ||||||
73340:      802 aaatttgtttcacaatcatttaaaggagaagcgttggcatggtggagatcattgctccaa 743


HAG001C01F: 241 gccacggggaagattctactctacaacttatcttggggccaatttgttgcattaatcaaa 300
                |||||||||||  ||||||||||||||||||||||||||||||||||||| ||| |||| 
73340:      742 gccacggggaaagttctactctacaacttatcttggggccaatttgttgctttagtcaag 683


HAG001C01F: 301 gaaaactattgccctcagcatgaagtagaaaagatagaatcggacttcctgacgttggtc 360
                ||||||||||||||||||||||||||||||||||||||||| |||||| |||| ||||||
73340:      682 gaaaactattgccctcagcatgaagtagaaaagatagaatcagacttcttgactttggtc 623


HAG001C01F: 361 atggaaaatttgaactgtcaggcatacgtgacaagtttcaacactatgtcccgcctagtc 420
                |||||||||||||| |||| |||||| |||||||||||||||||||||||||||||||| 
73340:      622 atggaaaatttgaattgtcnggcatangtgacaagtttcaacactatgtcccgcctagtt 563


HAG001C01F: 421 ccttatttagtcacccctgaacctaaacgcatagcgcgtttcattggaggcctagcacca 480
                ||||||||||||||||||||||||||||||||||||||||||||||||||||||||||||
73340:      562 ccttatttagtcacccctgaacctaaacgcatagcgcgtttcattggaggcctagcacca 503


HAG001C01F: 481 gagataaaaggaaatgttaaagcatctaggccaaccacatataggttcgcggtggactta 540
                |||||||||||||||||||||||||||||||||||||||||||||| |||||||||||||
73340:      502 gagataaaaggaaatgttaaagcatctaggccaaccacatataggtccgcggtggactta 443


HAG001C01F: 541 tccctatctctcaccttagatgcaatcagatttaagtctgttaaggcttct 591
                ||| |||||||||||||||||||||||||| ||||||||||||||||||||
73340:      442 tccttatctctcaccttagatgcaatcagaattaagtctgttaaggcttct 392

Query= HAG001C03F
Subject= 46146

HAG001C03F: 1 cctaacaacaaacaggccaatgataagatttacttaggaactaaaccaagacgtgctggg 60
              |||||||||| |||||||||||||||||||||||||  | ||||| ||||||||||||||
46146:     74 cctaacaacagacaggccaatgataagatttacttanaanctaaancaagacgtgctggg 133
```

```
HAG001C03F: 61 tgcaagtatcaccatgaatgtggatgtcggaagcctaggtgcgggaggaaaggacacaac 120
               ||||||||| |||||||||||||||||||||||||||||  |||||| ||| | ||||||
46146:     134 tgcaagtatnaccatgaatgtggatgtcggaagcctaggnncgggagnaaanggcacaac 193


HAG001C03F: 121 aaggatggttgtgggatgaagagtcccagacaaggagaaaacagtaacaacgctcaaggt 180
                |||||||||||||||||||||| ||||| ||| ||||||||   ||||||||||||||||
46146:      194 aaggatggttgtgggatgaagantcccanacanggagaaaancataacaacgctcaaggt 253


HAG001C03F: 181 ggaaatgggaaaggaaatggccgagtgccaggatgcttccattgtggggacgttgggcat 240
                ||||   ||||||||||||||||||||| |||||||||||||||||||||| |||| |||
46146:      254 ggaagcaggaaaggaaatggccgagtgctaggatgcttccattgtggggacattggacat 313


HAG001C03F: 241 cttaagagggaatgcccgaagttgaaacaagaccgtgaaagaacatccaacatcggagtt 300
                ||||||||||||||||||||||||||||||||||||||||||||| ||| ||||||||||
46146:      314 cttaagagggaatgcccgaagttgaaacaagaccgtgaaagaacacccagcatcggagtt 373


HAG001C03F: 301 tgtgaagcatgccaggatccaggcatgattactggtacgttctctaacaaccaaaacttt 360
                |||||||||||||||||||||||| |||||||||||||||||| ||||||||||||||||
46146:      374 tgtgaagcatgccaggatccaggcgtgattactggtacgttctttaacaaccaaaacttt 433


HAG001C03F: 361 tgcatctattttgtttgatactggtgccgatcatagcttcgtgtctctagaatttaagaa 420
                |||||||||||||||||||||||||||||||||||||||||||| |||||||||||||||
46146:      434 tgcatctattttgtttgatactggtgccgatcatagcttcgtgtcactagaatttaagaa 493


HAG001C03F: 421 tattcttggtttgacgcctagtaagtt-agacgtcccatattcgatcgaattaacaaatg 479
                ||||||||||||||||||||||||||| ||||||||||||||||| ||||||||||||||
46146:      494 tattcttggtttgacgcctagtaagttaagacgtcccatattcgagcgaattaacaaatg 553


HAG001C03F: 480 ggaaactagttgagaccagtgaaggtgtccaaggatgtgtgatggaatttggagggcatg 539
                | |||||||| ||||||||||||| |||||||||||||||||||||||| |||||||| |
46146:      554 gaaaactagtggagaccagtgaagttgtccaaggatgtgtgatggaattcggagggcacg 613


HAG001C03F: 540 agtttacgctagacttactacctgtcgagttgggaagctttgatgtggtagtgggaacgg 599
                ||||||||||| ||||||||| ||| ||||||||||||  || |||||||| |||| ||
46146:      614 ggtttacgctaggcttactaccagtccagttgggaagctacgangtggtagtaggaatgg 673


HAG001C03F: 600 attagctatcgagtaaacaggccaagattgtttgtcgcgaaaaggtcgttcgtatccc 657
                ||| | | ||  | ||  ||||| |||| |||||||  || |||||||||||||||||
46146:      674 attggttgtccggaaacaaggccgagatagtttgtcatgagaaggtcgttcgtatccc 731
```

Query= HAG001C09F
Subject= 42764

```
HAG001C09F: 1 atggattgcaagcctctccctttcaatggcactgagggtgccataggtcttctgcaccgc 60
              ||||||||||| ||||| || |||| |||||||||||||||||||||||| |||||| |
42764:   1391 atggattgcaancctcttccattcagtggcactgagggtgccataggtctnctgcactgg 1332


HAG001C09F: 61 attgagaaggtcgaagctgttttcgctgtctaagagtatcccccagctaattgggtgaag 120
               || ||||| || |||||||| || |||||||  |||| |||||| || ||||||||||||
42764:    1331 atngagaangtngaagctgtcttngctgtctgngagtgtccccctgcnaattgggtgaag 1272
```

```
HAG001C09F: 121 tttgctattggtacacttgaaggaaacgcgctttcatggtggaaagcgcagattcaaatg 180
                ||||||| |||||| || || |||| ||| ||||| |||||||| ||||| |||||||||
42764:     1271 tttgctactggtactctngagggaagcgcactttcntggtggaaggcgcaaattcaaatg 1212


HAG001C09F: 181 cttggtttggaaactgccaatgctactgcatgggaagatttcaaggacatgatcaaggaa 240
                ||||||||||| | ||| || |||||||||||||||||||||||||| ||||||||||||
42764:     1211 cttggtttggagantgctaangctactgcatgggaagatttcaaggatatgatcaaggaa 1152


HAG001C09F: 241 gagtactgtcacagggatgacatttacaaactcgaaaatgagtactttgagctcaagatg 300
                ||||| |||||||||||||||||  ||||||||||  | ||||||| ||| |||||||||
42764:     1151 gagtantgtcacagggatgacatccacaaactcgaggacgagtactatgaactcaagatg 1092


HAG001C09F: 301 gttggatcagagatcgagacctacaccaaatagtccaacgactatgctgctctgtgccca 360
                 |||| |||||||| ||||||||||||||   ||||||||||||||||||||| || |||
42764:     1091 nttgggtcagagattgagacctacaccaagctgtccaacgactatgctgctctttgncca 1032


HAG001C09F: 361 aacatgtctcgacctatgtatcgaaggattgaattgtacatcaagggtttggccccagaa 420
                |||||||| ||||| ||||| |||||||| |||||||||||||| || || | ||||||
42764:     1031 aacatgtcccgaccnatgtaccgaaggatcgaattgtacatcaaaggcttagtcccagag 972


HAG001C09F: 421 attcggagccatgtgacctcagccaacctcaataccattcagccagtcgtccgtcttgct 480
                ||  |||||||||||||||| |||||||||| |||||| |||||  | || || |||||
42764:      971 atcaggagccatgtgacctcggccaacctcactaccatacagccnntngttcgncttgcn 912


HAG001C09F: 481 cacaaactcactgatcaggctgtggaacagggcaagttgcccaaaaggatcagtgctact 540
                |||||||||||  | ||||| |||||| |||||  |||||||||||||||||||| | |
42764:      911 cacaaactcacnnancaggcngtggaagagggcnngttgcccaaaaggatcagtgntncn 852


HAG001C09F: 541 gctggaacttctggtgataacaagcgtaagtgggaaggaaaccaaaacaaggatgctaac 600
                |  ||||||||| ||||   ||| || |||||||| ||||  ||   |||||||||||||
42764:      851 gnnggaacttctagtganngcaancgnaagtggganggaantcanngcaaggatgctaac 792


HAG001C09F: 601 cccactcaggtcccatctcagcaaaggaaaactgaaaacaacaagggccctcagcaacag 660
                |||||||||| |||| |||||||||||||||| ||||||||||| ||| |||| ||||||
42764:      791 cccactcaggccccagctcagcaaaggaaaacngaaaacaacaaaggcactcancaacag 732

HAG001C09F: 661 ggtggcta 668
                ||||||||
42764:      731 ggtggcta 724


Query= HAG001C21F
Subject= 218181
HAG001C21F: 1   attgttagattggcacataagatcgttgaccaggaggtggagagcgactcattgccacct 60
                |||||||||||||||||||| |||||||||||||||||||||||||||||||||||||||
218181:     151 attgttagattggcacataaaatcgttgaccaggaggtggagagcgactcattgccacct 210


HAG001C21F: 61 cgtatttctagcactaccacagctgccactacttccactgcatctgctgctgatagcaaa 120
               ||||||||||||||||||||||||||||||||||||||||||||||||||||||||||||
218181:    211 cgtatttctagcactaccacagctgccactacttccactgcatctgctgctgatagcaaa 270


HAG001C21F: 121 cgtaagtggaacgatacggacaaggggtccaactctgcacagcctcagaagaagacggat 180
                |||||||||||||||||||||||||||||||||||| |||||||||||||||||||||||
218181:     271 cgtaagtggaacgatacggacaaggggtccaactctacacagcctcagaagaagacggat 330
```

```
HAG001C21F: 181 actggcagcacccgcagttccagtcagacagcatcagtgaatcagaactcgagtaacaaa 240
                ||||||||||||||||||| |||||||||||||||||||||||||||||||||||||| |
218181:     331 actggcagcacccgcagtttcagtcagacagcatcagtgaatcagaactcgagtaacaga 390

HAG001C21F: 241 tcagggcagggatcatatgcggggaagctacccttgtgcagcaagtgtgactatcatcac 300
                ||||||||||||||||||||||||||||||||||||||||||||||||||   |||||||
218181:     391 tcagggcagggatcatatgcggggaagctacccttgtgcagcaagtgtgatcttcatcac 450

HAG001C21F: 301 aagggacagtgtgctcgggtttgtcatcgatgtaatcgaccagggcatatggctagggat 360
                ||||| |||||| |||| |||||| |||| ||||||||||||||||| | ||| ||||||
218181:     451 aaggggcagtgtactcgagtttgttatcggtgtaatcgaccagggcacaaggccagggat 510

HAG001C21F: 361 tgtagggccacttttccagctcagcagcagccatcacagcagatgggtaggcaacaatct 420
                |||||| ||||  |||||||||||||||||||| | ||||||  ||||||||||||  ||
218181:     511 tgtaggaccacacttccagctcagcagcagccagcgcagcaggcgggtaggcaacagcct 570

HAG001C21F: 421 caacaaaatcagggtactcagagagggtgttatcagtgtggagccgaggggcacttcaag 480
                || |||| |||||||| ||||| |||||||||||||||||| ||||||||||||||||||
218181:     571 cagcaaactcagggtaatcagaaagggtgttatcagtgtggggccgaggggcacttcaag 630

HAG001C21F: 481 cgagactgccctcaactgaagcagaatacaggggggtggtaacgggaacaacaatgcgggg 540
                |||||||||||||| ||||  ||||| ||||||||| |||||||||||||||||||||||
218181:     631 cgagactgccctcagctganncagaacacaggggggtngtaacgggaacaacaatgcgggg 690

HAG001C21F: 541 aacaatgcgggtaatgtcgcgcgtgggcgcgggtttgtgttgggagc-tggtgaagcgcg 599
                || || | ||||||||| ||||||||||||||||||||| ||||||| ||| ||||||||
218181:     691 aanaangggggtaatgttgcgcgtgggcgcgggtttgtgctgggagcgtggggaagcgcg 750

HAG001C21F: 600 gaacgacggcaatgtggttactggtacgttttcagtgaacggtgttattgcttctatttt 659
                ||||||||||||| |||||||| ||||||||||| || ||||||||||||||||||||||
218181:     751 gaacgacggcaacgtggttacnggtacgttttcngtnaacggtgttattgcttctatttt 810

HAG001C21F: 660 atttgattctggtgccgactggagttacgtgtcttt 695
                ||||||||||||||||||||||||||||||||||||
218181:     811 atttgattctggtgccgactggagttacgtgtcttt 846
```

Query= HAG001E19F
Subject= 65097

```
HAG001E19F: 1 cgacatcaaatagatcgagctcttacatgtttctattcatctattgctgatatgcagaaa 60
              |||||||||||||| | ||||||||||||||||||||||||||||| ||||||||| |||
65097:    547 cgacatcaaatagaccaagctcttacatgtttctattcatctattgttgatatgcaaaaa 606

HAG001E19F: 61 gaagtctcattaaaatgttttatttcgtagatagagcccagaacctacaaagaggcattg 120
               || |||||||||||||||||||||||| |||| ||||||||||| |||||||||||||||
65097:     607 ga-gtctcattaaaatgttttatttcgcagatcgagcccagaacttacaaagaggcattg 665

HAG001E19F: 121 actgaggatagttgggtgaatgcaatgcaggaggagctacaacagttcga--aaagctgg 178
                ||| ||||||| ||||| ||||| |||||||||||||| ||||| || ||  ||||||||
65097:      666 actaaggatagctgggtaaatgcgatgcaggaggagctgcaacaatttgacaaaagctgg 725
```

```
HAG001E19F: 179 gcgtcttgagacttgtcgatctgccaaaaaat-cagaagttaat--caagacgaaatggg 235
                | |||| |||||| |||||||||||  ||||| || |||||||   ||| || || ||||
65097:      726 gtgtctggagactcgtcgatctgcctgaaaatacaaaagttaaatacaaaacaaagtggg 785


HAG001E19F: 236 tttttaagtgtaagcgtgatgacagaggtgtcgtggtgagaaacaaagcgcgacttgtgg 295
                |||||||||| || || ||||| |||||||||||| ||||||||||||||||||| ||||
65097:      786 tttttaagtgcaaacgagatgatagaggtgtcgtgatgagaaacaaagcgcgactagtgg 845


HAG001E19F: 296 ttcaaggcttcagtcaacaggaaggaatagattatgatgaagtctacgcgccggttgcca 355
                | ||||| || |||||| | ||||| || || ||||||||||||||||||||||||
65097:      846 tacaagggtttagtcaataagaaggcatcgactacgatgaagtctacgcgccggttgcca 905


HAG001E19F: 356 gactcgaggcaattcggatatttctagcctttgcgtcttggaaagattttaaagtatatc 415
                ||||||||||||||||||| ||||||||||| || ||||||||||| |||||||| || |
65097:      906 gactcgaggcaattcggatctttctagccttcgcttcttggaaagactttaaagtgtacc 965


HAG001E19F: 416 aacttgatgtcaaatctgccttcctttacggaacaatcagagaagaagtgtatgtggggc 475
                | || ||  ||||||| ||| ||||||| |||| ||||| ||||||||||||||||||||
65097:      966 agctggacatcaaatccgccatcctttatggaaaaatcaaagaagaagtgtatgtggggc 1025


HAG001E19F: 476 aaccgccggggttcacagaccctctgcacagaaacaaggtgtatctactggacaaagcgc 535
                |||||||||||||||| || || |||| || ||||||||| ||||| |||||||||||||
65097:     1026 aaccgccggggttcactgatccactgcccaaaaacaaggtctatcttctggacaaagcgc 1085
HAG001E19F: 536 tatacggacttcaccaggccccgagagcctggtacaagacgctttcgacatacttgttgg 595
                | || ||||||||| ||||||||||||||| |||| ||||||||||     |  || ||
65097:     1086 tctatggacttcactaggccccgagagcctagtacgagacgctttcccagcatctgctga 1145


HAG001E19F: 596 acaacgggttcacaagaggcacagtagacagcactttgttcaccaaggaagaagcaggcc 655
                 |||||||||||| ||||| |||||||| ||||| |||||||| |||||||  ||||| |
65097:     1146 ccaacgggttcaccagagggacagtagatagcacattgttcacaaaggaagtggcaggtc 1205


HAG001E19F: 656 acttgttgatcgtgcagatatatgttgacgatatcatctttggatccaccaacgacga 713
                | ||||||||||||||||| ||||||||||||||||| || || ||||||||||||||
65097:     1206 atttgttgatcgtgcagatntatgttgacgatatcattttcggttccaccaacgacga 1263


Query= HAG001G05F
Subject= 44213
HAG001G05F: 1 taatgtctatcaccggagggacatttggaacgaattatgagaatgaagattgggagtttt 60
              ||||||||||||||||||||||||||||||||||||||||||||||||||||||||||||
44213:   3295 taatgtctatcaccggagggacatttggaacgaattatgagaatgaagattgggagtttt 3236


HAG001G05F: 61 tggagaatatggcaactacatcaaaaaggaaagcccaagcttcgaggagagcccgaccga 120
               |||||| |||||||||||||||||||||||||||||||||||||||||||||||||||||
44213:    3235 tggagagtatggcaactacatcaaaaaggaaagcccaagcttcgaggagagcccgaccga 3176


HAG001G05F: 121 caactaaccgaccacaagtgcatgccatagatgaaggtaatgttcaaagtactaaccaaa 180
                ||||||||||||||||||||||||||||||||||||||||||||||| ||||||||||||
44213:     3175 caactaaccgaccacaagtgcatgccatagatgaaggtaatgttcaaactactaaccaaa 3116


HAG001G05F: 181 tttatgatgtgtgtgctttgtgtaatgaaataggtcacgcggctgaaaattgccaaggga 240
                ||||||||||||||||||||||||||||||||||||| |||||||||||||||||||| |
44213:     3115 tttatgatgtgtgtgctttgtgtaatgaaataggtcangcggctgaaaattgccaaggaa 3056
```

```
HAG001G05F: 241 tgttggaagggcaatatgaggaagttcatgcgatccaaggtcaaggccaaggaggaggtg 300
                ||||||||||||||||||||||||| |||||| | ||||||||||| |||||||||||||
44213:     3055 tgttggaagggcaatatgaggaagtccatgcggttcaaggtcaaggtcaaggaggaggtg 2996


HAG001G05F: 301 gtaggaactacaacaacatgaattctaatacctaccaccccgggttgaggaatcacccga 360
                ||||||||||||||||||||||||||||||||||||||||||||||||||||||||||||
44213:     2995 gtaggaactacaacaacatgaattctaatacctaccaccccgggttgaggaatcacccga 2936


HAG001G05F: 361 actttagatatgggaacccgtcaaatcaagcgaacccaaattttcaaggtagccaaggta 420
                ||||||||||||||||||||||||||||||||||||||||||||||||||||||||||||
44213:     2935 actttagatatgggaacccgtcaaatcaagcgaacccaaattttcaaggtagccaaggta 2876


HAG001G05F: 421 atttggttcacggccatcttataataaccaaggtgggtaccggggcggaaataaccaag 480
                ||||||||||||||||||||| |||||||||||||||||||| |||||||||||||||||
44213:     2875 atttggttcacggccatcttanaataaccaaggtgggtaccgnggcggaaataaccaag 2816

HAG001G05F: 481 ggtatcaaaaacaataccaaacgggtcaagagcaaaggggatcttcgggtggaaacgagg 540
                ||||||||||||||||||||||||||||||| ||| |||| |||||||||||||| ||||
44213:     2815 ggtatcaaaaacaataccaaacgggtcaagancaangggntcttcgggtggaaangagg 2756


HAG001G05F: 541 tgatggagatgcttaaaagcatgcaattggaaatgcaaaagcggaatcaacttgatgaag 600
                ||||||||||||| || ||||||||| |||| |||||||| ||||| |||||||||||||
44213:     2755 tgatggagatgctnaanagcatgcaantgganatgcaaaancggaancaacttgatgaag 2696


HAG001G05F: 601 tgcggatgcaaaaagatgaggttcgtgataaaagcattcagtcactaacgactcaaatgg 660
                |||| |||||||||||||||||||| ||||||||||| || |||||||| || |||||||
44213:     2695 tgcgnatgcaaaaagatgaggttcgngataaaagcatccantcactaacaacccaaatgg 2636


HAG001G05F: 661 g 661
                |
44213:     2635 g 2635


Query= HAG001G21F
Subject= 46419

HAG001G21F: 1 tcctcgggatgaggttcagaagctggaaaacgagtactatgacttgaagatggttggatc 60
              |||||||||||| || |||||| |||||||||||||||| ||||||||||||||||||||
46419:    759 tcctcgggatgaagtccagaagttggaaaacgagtactacgacttgaagatggttggatc 700


HAG001G21F: 61 tgaagtcgaagcgtatatgaagcgatcgtatgaattggcggacatgtgcccgaacttgtc 120
               |||||||||||||||| ||||||||||||||||| |||| ||||||||||||||||||||
46419:     699 tgaagtcgaagcgtatgtgaagcgatcgtatgaactggccgacatgtgcccgaacttgtc 640


HAG001G21F: 121 ccggcctatgtctcggaggattgagttattcatcaaggggttgcctccgcgtgtgaagag 180
                |||||||||||||||||||||||||||||||||||| |||||||||||||||||||||||
46419:      639 ccggcctatgtctcggaggattgagttattcatcaaagggttgcctccgcgtgtgaagag 580


HAG001G21F: 181 tttggtcactgcagcccatctcaatgatttgacacagattgttcgattgacccacaagat 240
                ||||||||||||||||||||||||||||||||||||||| |||||  |||| ||||||||
46419:      579 cttggtcactgcagcccatctcaatgatttgacacagatcgttcgtctgactcacaagat 520
```

```
HAG001G21F: 241 tgtggatcaagaggtagagagtaactcgttaccgccgcgtgtttcaaccactactgctgc 300
                ||||||||||||||| || ||  | |||||||| |||||| ||||  || ||||||||||
46419:      519 tgtggatcaagaggtggaaagcgattcgttaccnccgcgtatttcggccnctactgctgc 460


HAG001G21F: 301 tgcacccactgccactgcgtccgctaatgataacaaaaggaagtggagtgactatgataa 360
                | ||||||||||||||||||||||||||||||||||  | |||||||||||  |||| ||
46419:      459 tacacccactgccactgcgtccgctaatgataacaagcgnaagtggagtgatnatgacaa 400


HAG001G21F: 361 agcctctagcgcgagtcagacacagaaaagaccagataataacaacaaccgcagcatcag 420
                ||| ||  | ||  | ||||| |||||||| ||||   | |||| || ||||| ||||||
46419:      399 agcatcnngtgctggccagactcagaaaaggccag---acaacagcanccgcaacatcag 343


HAG001G21F: 421 ccagtcgtcttctgtcaatcaaggccaagggggtagccaaagccagggtcagtacgcggg 480
                |||||||||||||||||||| |||||| ||  |   ||| |||||||||  ||| || ||
46419:      342 ccagtcgtcttctgtcaatcnaggccagggaagngnccacagccagggttcgtatgcagg 283


HAG001G21F: 481 gaggaagccacgatgtaacaagtgtggctatcatcatttcgggccgtgtggttggatatg 540
                ||||||||||||||| |||| |||||||||||||||||  ||||||||| || |||  ||
46419:      282 gaggaagccacgatgnaacangtgtggctatcatcattnngggccgtgtngtcggacgtg 223


HAG001G21F: 541 taacaggtgtggtaaggcgggccatgaggccagggattgtagggccccacagcccanaca 600
                |||||  ||||||||| | ||||||||||||||||||||||||||||||||||||| |||
46419:      222 taacanntgtggtaagncnggccatgaggccagggattgtagggccccacagcccaaaca 163


HAG001G21F: 601 ccagcagcaacagaaccagcaggaccagagacagcagggacaactaccccagcagaatca 660
                |||||||||||||||||||||| | |||||||| |||||||||| |||||||||||| ||
46419:      162 ccagcagcaacagaaccagcagaancagagacancagggacaaccaccccagcagaanca 103


HAG001G21F: 661 gggcttcaggaaggggtgctatcagtgtggagacgagggtcactttaagcgggattgccc 720
                ||| |||||||||||||||||||||||||| |||||||||||||||||||||||||||||
46419:      102 gggnttcaggaaggggtgctatcagtgtggtgacgagggtcactttaagcgggattgccc 43


HAG001G21F: 721 tcagtt 726
                ||||||
46419:       42 tcagtt 37
```

Query= HAG001I11F
Subject= 93740

```
HAG001I11F: 1 atctctataatgtaaatgattatgatggctatagattaaatattaatacgtaatatattt 60
              |||||||||||||||||||||| |||||||||||||||||||||||||| ||||||||||
93740:    708 atctctataatgtaaatgattangatggctatagattaaatattaatacataatatattt 649


HAG001I11F: 61 aatcttacattctcccacttagccgagtaatcatttactatgagtattagataagcctga 120
               ||||||||||||||||||||||||||||||||||||||||||||||||||||||||||||
93740:     648 aatcttacattctcccacttagccgagtaatcatttactatgagtattagataagcctga 589


HAG001I11F: 121 tcaggagttaacactcatttagccttaaacaagtactatagcagaaaaatacagccgtt 180
               ||||||||||||||||||||||||||||||||||||||||||||||||||||||||||||
93740:     588 tcaggagttaacactcatttagccttaaacaagtactatagcagaaaaatacagccgtt 529
```

```
HAG001I11F: 181 gaatcattatgcgactcaggacccccattaatcatactatagccttaatttaaaacctag 240
                ||||||||||||||||||||||||||||||||||||||||||||||||||||||||||| 
93740:      528 gaatcattatgcgactcaggacccccattaatcatactatagccttaatttaaaacctaa 469


HAG001I11F: 241 tcgtcatgatcaaaatacgcgtaagccctttgtttgatttgtaacttttattttgtctat 300
                ||||||||||||||||||||||||||||||||||||||||||||||||   |||||||||
93740:      468 tcgtcatgatcaaaatacgcgtaagccctttgtttgatttgtaacttt--atttgtctat 411

HAG001I11F: 301 atgccattataccggttgaacatattctaacagacgtacagaatcaaacttgaggaaatt 360
                |||||||||||||||||||||||||||||| |||| |||| |||||||||||||||||||
93740:      410 atgccattataccggttgaacatattctaagagacatacaaaatcaaacttgaggaaatt 351


HAG001I11F: 361 tcgttaatcataaaacataagctagtacattatgctcaaataaggtctttactaaatccc 420
                |  |||| ||||||||||||||||||||| |||||| |||||||||||||||||||||||
93740:      350 tnattaa-cataaaacataagctagtacaatatgcttaaataaggtctttactaaatccc 292


HAG001I11F: 421 atattccgaacatgttcttcgtaaaccttaggagggagacctttagtcatcggatccgca 480
                ||||||||||||||||||||||||||||||| ||||||||||||||||||||||||||||
93740:      291 atattccgaacatgttcttcgtaaaccttaggngggagacctttagtcatcggatccgca 232


HAG001I11F: 481 agcatatccttagtactaatatactcgatacaaagattattttcctcaactcgttcacgt 540
                ||||||||||||||||||||||||||||||||||||||||||||||||||||||||||||
93740:      231 agcatatccttagtactaatatactcgatacaaagattattttcctcaactcgttcacgt 172


HAG001I11F: 541 acaaatagatatttcgtatcgagatataaaccagctccagtcgaactgttactgttcgag 600
                ||||||||||||||||||||||||||||||||||||||||||||||||||||||||||||
93740:      171 acaaatagatatttcgtatcgagatataaaccagctccagtcgaactgttactgttcgag 112


HAG001I11F: 601 aaactaacggcagctgaattatcacagtagagcttcaatggtctagaaatg 651
                ||||||||||||||||||||||||||||| |||||||||||||||||||||
93740:      111 aaactaacggcagctgaattatcacagtaaagcttcaatggtctagaaatg 61


Query= HAG001K01F
Subject= 47464
HAG001K01F: 1 catgaagtgacacttctgccaattcaacataagttttaactcgacacaccgcttaaacat 60
              ||||||||||||||||| |||||| | |||||| ||   ||| |||||||||||||||||
47464:   1120 catgaagtgacacttctcccaatttagcataagcttcgtctctacacaccgcttaaacat 1061


HAG001K01F: 61 cttgttgagattcttcaaacactcatcaaaggagttgccatataccgaaaaatcttccat 120
               | | |  | ||||||||||||| |||| || || |  ||||| || |||||||| |||||
47464:    1060 cctttccaaattcttcaaacacacatcgaaagaatcaccataaactgaaaaatcatccat 1001


HAG001K01F: 121 gaaaacctccatggaactctcaatcatatcctgaaatatggcaaccatgcatcgctgaaa 180
                |||||||||||||||| |||| |||||||||||||||||||| | |||||| || |||||
47464:     1000 gaaaacctccatggaagtctctatcatatcctgaaatatggcnancatgcaacgntgaaa 941


HAG001K01F: 181 ggtagctggagcgttacatagcccaaatggcatgcgtcggaacgcataggtgccataggg 240
                 ||||||||||||||||||| ||||||||||||||||||| |||||||||||||||||||
47464:      940 agtagctggagcgttacatancccaaatggcatgcgtcggtacgcataggtgccataggg 881


HAG001K01F: 241 gcatgtaaaagtggtcttatcctgatcctccagtgcgatggggatctggaagtacccgga 300
                |||||| || ||||||||||||||||||||| |||||||||||||||||||||| || ||
47464:      880 gcatgtgaaggtggtcttatcctgatcctccngtgcgatggggatctggaagtanccaga 821
```

```
HAG001K01F: 301 aaaaccgtcgagaaaacaatagaattgttgacccgcgagacgctctaacatttgatcgat 360
                ||| ||||| ||||||||||| ||||||||||| ||||||||||| ||||||||||| ||
47464:      820 aaanccgtcnagaaaacaatanaattgttgaccngcgagacgctccaacatttgatcnat 761


HAG001K01F: 361 gaagggcaatgggaagtggtcttttcgggtggcatcatttaactttcagcagtcgatgca 420
                ||| ||||||||||||||||||||||||||||||||||| ||||||| | ||||||||||
47464:      760 gaanggcaatgggaagtggtcttttcgggtggcatcattcaactttcggtagtcgatgca 701


HAG001K01F: 421 catgagccaatccgtgaccgtacgagaagggataaactcattcttagaattcataacaac 480
                || | ||||| |||||||||||||||||||||||| ||| |||||    ||||| |||||
47464:      700 cacgcgccaacccgtgaccgtacgagaagggataagctcgttcttttcgttcatgacaac 641


HAG001K01F: 481 ggtcatcca---tcccttcttagtgaaaacctgagtaggactcacccaaggtgaatcgga 537
                ||||||||     |||||||| | || ||||||||| ||||| |||||||||||||| ||
47464:      640 ggtcatccncccccccttcttggggacaacctgagtgggactaacccaaggtgaatcnga 581


HAG001K01F: 538 aatgggatatatcatcccggcatctaggagtttaagaacctccttcttcacaacatcttg 597
                ||||||||| |||||||| |||||||||||||||||||||||||||||||| ||||||||
47464:      580 aatgggatagatcatcccngcatctaggagtttaagaacctccttcttcactacatcttg 521


HAG001K01F: 598 catgtt 603
                ||||||
47464:      520 catgtt 515
```

Query= HAG001M15F
Subject=67655

```
HAG001M15F: 1 atgagacaccaccttcatattcttattatgaggaaccatggcgtgaacaacccacctcat 60
              ||||| ||||||||||||| ||||||||||||||||||||||||||||||||||||||||
67655:   1316 atgagccaccaccttcatactcttattatgaggaaccatggcgtgaacaacccacctcat 1257


HAG001M15F: 61 atgagtactatgaagaacaaagtttcgacccttatccatcatacacttacaatgaagaac 120
               ||||||||||||||||||||||||||||||||||||||||||| ||||||||||||||||
67655:    1256 atgagtactatgaagaacaaagtttcgacccttatccatcatatacttacaatgaagaac 1197


HAG001M15F: 121 aatggtgcgaatcatctacttcatatgagtactatgaggaaccaaggatcgaacaaccga 180
                ||||||| ||| |||||||||||||||||||||||||||||||||||||||||||||||
67655:     1196 aatggtgtgaaccatctacttcatatgagtactatgaggaaccaaggatcgaacaaccgg 1137


HAG001M15F: 181 attcaagcttttgaggatccaaattctttcaatctcaccgaagtaactaataggataata 240
                ||||||||||| ||||||||||||||||||  |||||||||||| || ||||||||| ||
67655:     1136 attcaagcttt-gaggatccaaattctttctctctcaccgaagtgaccaataggatatta 1078


HAG001M15F: 241 gaacacattaaaactatccaacgttgcataaaagaatctcgcgcaagggaagaggaatcc 300
                |||||||||||||||||| ||||||||||||||||||||||||||||||||||||| |||
67655:     1077 gaacacattaaaactatcgaacgttgcataaaagaatctcgcgcaagggaagaggagtcc 1018


HAG001M15F: 301 cgcgcaagagaagagttaaaaaatgataataacgtagagatagttgaaagtgtaaaaatg 360
                |||||||||||||| |||||   | |||||||||||||||||||||||| ||||||||||
67655:     1017 cgcgcaagagaagaattaaattgtaataataacgtagagatagttgaaaatgtaaaaatg 958
```

```
HAG001M15F: 361 gaagaacaagaaagtgaaaaacagacacatgagttaaacaacgaaaatggtgagtccgat 420
                |||||||||||||||||||||| |||||||||||||||||||||||||||||||||||||
67655:      957 gaagaacaagaaagtgaaaaaccgacacatgagttaaacaacgaaaatggtgagtccgat 898


HAG001M15F: 421 aatgttaaaattcaagaagagtctaattttgaggaaattattctcttgtcacctactttc 480
                |||||||||||||||||||||||||||||||| ||||||| |||||||||||||||||||
67655:      897 aatgttaaaattcaagaagagtctaattttgaagaaattaatctcttgtcacctactttc 838


HAG001M15F: 481 gaaaatcattgtttaataacccctcatgccaagtttttaaaagagttaaacactaatgct 540
                ||||||||||||||| ||||||||||||||||||||||||||||||||||||||| ||||
67655:      837 gaaaatcattgtttantaacccctcatgccaagtttttaaaagagttaaacactagtgct 778


HAG001M15F: 541 aaaatcaatgacttagtaagtgttaagttaactaatgatcaaacctcgctaataaaagaa 600
                |||||||| || ||||||||||||||||||||||||||||||||||||||||||||||||
67655:      777 aaaatcaaaganttagtaagtgttaagttaactaatgatcaaacctcgctaataaaagaa 718


HAG001M15F: 601 gatccttttgaaattaacattacaccggttccatgtttctttcaaaattcgtttatttag 660
                |||||||||||||||||||||||||||||||||||||||||||||||||| ||||||
67655:      717 gatccttttgaaattaacattacaccggttccatgtttctttcaaaattcatttattagt 658


HAG001M15F: 661 catatcactattgataangatctttgt 687
                 ||||||| |||||||| |||||||||
67655:      657 aatatcaccattgataaagatctttgt 631



Query= HAG001O17F
Subject= 67629

HAG001O17F: 1 cccataaccaggcccaaatcatttgcggcaagcggcaaatagttttaaagactccgagtg 60
              |||||||||||||||||||||| ||||||||  |||| ||||||||||||||||||||||
67629:    916 cccataaccaggcccaaatcatctgcggcaaaaggcagatagttttaaagactccgagtg 857


HAG001O17F: 61 gtgaatctcttaccattcgaggggatacgcattacggattgcccgaagacgtatctatgc 120
               ||||||||||||||||||||||||||||||||||||||||||||||||||||||||||||
67629:     856 gtgaatctcttaccattcgaggggatacgcattacggattgcccgaagacgtatctatgc 797


HAG001O17F: 121 tgaaagcttcaaggtgtttgaacagaggctgtgtaatttacatggctcaggtgataattg 180
                ||||||||||||||||||||||||||||||||||||||||||||||||||||||||||||
67629:      796 tgaaagcttcaaggtgtttgaacagaggctgtgtaatttacatggctcaggtgataattg 737


HAG001O17F: 181 aagaaccaaggccgaagatcgaggatcttcctgtcatttctgaataccccgaggtttttc 240
                ||||||||| |||||||||||||||||||||||||||||||||||| ||||||||||| |
67629:      736 aagaaccaaagccgaagatcgaggatcttcctgtcatttctgaatancccgaggttttnc 677


HAG001O17F: 241 ctgaagaactacctggtttgccaccagatagacaagtggagttcagaattgacatcattc 300
                | |||||||||||||||||||||||||||||||||||||||||||||||||||||||| |
67629:      676 cngaagaactacctggtttgccaccagatagacaagtggagttcagaattgacatcatnc 617


HAG001O17F: 301 ctggagcagctccgatagcacgagcaccttacagattagcgccaacggaaatgaaagaac 360
                |||||||||| || || ||  |||||||||||||  |||| |||||||||||||||||||
67629:      616 ctggagcagcnccnatngcnagagcaccttacagnntagcaccaacggaaatgaaagaac 557
```

```
HAG001O17F: 361 tgaggacccagttggatgaactgctggcgaaaggttttatcagacctagttcatctcctt 420
                ||||||||||||||||||||||||| || |||||||| |||| ||||||||| ||||| |
67629:      556 tgaggacccagttggatgaactgctngcnaaaggtttnatcanacctagttcntctccct 497


HAG001O17F: 421 ggggagcaccggtcctttttgtaaagaagaaggacgggtcaatgcgtttatgcattgatt 480
                |||||||||| || || | ||| |||||||||||||| || |||||  | ||||| ||||
67629:      496 ggggagcacctgtnctgtntgtcaagaagaaggacggatcgatgcgnctgtgcatcgatt 437


HAG001O17F: 481 atagagagctgaataaagttaccataaagaatagatatcctttaccaaggatcgatgatc 540
                |  | ||||| ||||| || || |||||||||||||||||||||||||||||||||||||
67629:      436 ancgngagcttaataaggtcacgataaagaatagatatcctttaccaaggatcgatgatc 377


HAG001O17F: 541 tattcgatcagctgcaaggagcaagctacttctccaagatcgacttaaggtcgggttatc 600
                ||||||||||||||||||||||||||||||||||||||||||||||||||||||||||||
67629:      376 tattcgatcagctgcaaggagcaagctacttctccaagatcgacttaaggtcgggttatc 317


HAG001O17F: 601 atcaactaagggtcagagatgaagatgtacacaagactgcatttaggactcgctatggtc 660
                ||||||||||||||||||||||||||||||| || ||  |||||||||||||||||||||
67629:      316 atcaactaagggtcagagatgaagatgtacanaanacnncatttaggactcgctatggtc 257


HAG001O17F: 661 attacgagttcctagtgatgccttttgggctcacaantgcaccggctgcgttcatggatc 720
                ||||||||||||||||||||||||||||||||||||| |||||||||||| ||||||||||
67629:      256 attacgagttcctagtgatgccttttgggctcacaaatgcaccggctgcattcatggatc 197

HAG001O17F: 721 t 721
                |
67629:      196 t 196


Query= HAG002A15R
Subject= 67322

HAG002A15R: 1 atagatcatgtgacgtatagggtgccataagcaccattaatcaaatacatacctagaccg 60
              ||||||||||| ||| |||||||| |||||||||||| ||||||    |  |||||||||
67322:    887 atagatcatgtcacgaatagggtgtcataagcaccatcaatcaacgtaagtcctagaccg 828


HAG002A15R: 61 cggaacaaaatgttagatctaatgggtttcaggcaaccacactcttggccacacttttac 120
                ||||||||| |||||||||||||||||||||||| ||||||||||||||||| ||||||
67322:     827 tggaacaaaaggttagatctaatgggtttcaggcagccacactcttggccacatttttac 768

HAG002A15R: 121 caaaaagtgcctttgtgtctctagtcgtgaatcgacaagtaaaaatgcctatgggtttgg 180
                ||  | |||| |||||||||||||||||||||||||||||||||| |||||||| |||||
67322:      767 catga-gtgcttttgtgtctctagtcgtgaatcgacaagtaaaa-tgcctatgg-tttgg 711


HAG002A15R: 181 atgcttcctatgtcgttacacatgttaatggccttgcaaaccattagcgatctcgattta 240
                |||||||||||||||||||| |||||||||||||||||||||||||||||||||||||| 
67322:      710 atgcttcctatgtcgttacatatgttaatggccttgcaaaccattagcgatctcgatttc 651


HAG002A15R: 241 cttacatttacagaagtacttatttca-gtacaattacataccatgttttctacagcgaa 299
                ||||||||||||||| ||||  ||||| |||||||||||||||||| ||  |||| ||||
67322:      650 cttacatttacagaaatact--tttcangtacaattacataccatgnttcntacaacgaa 593


HAG002A15R: 300 tgcttaactgttttatacaaaactgcatgaattcacaccaacattatgttgacgatttgc 359
                | ||||| ||||||   |||||||| |||| ||| ||||||| ||||||||||||||| |
67322:      592 tacttaaatgttttncncaaaactgnatganttcncaccaac-ttatgttgacgatttnc 534
```

```
HAG002A15R: 360 caaaacttacatgtatt 376
                |||||||||||||||||
67322:      533 caaaacttacatgtatt 517


Query= HAG002A23R
Subject= 43451

HAG002A23R: 1 tgcctctgtggtgcgccacgtttataagggtccttggctagacccaatacgaggttatat 60
              |||||| ||||||||||||||||||||||||||||||||||||||||| |  ||| | |
43451:     67 tgcctccgtggtgcgccacgtttataagggtccttggctagacccaattcccggtcacac 126


HAG002A23R: 61 gt--tttctgagtgggatgcttggcgtcacatgttacaccgtcggagagcagcatccaag 118
               ||  ||||||| |||| ||| | || || || |||||||||||||||||| |||||||||
43451:     127 gtcatttctgattggggtgccttgcctcccaagttacaccgtcggagagcggcatccaag 186


HAG002A23R: 119 cttgaatcaataaccttcatgtaattgaccgggtcgtcgtcctctattctcttcccaact 178
                ||||| || |||||||||||||| | |||||||||||| ||||||||||| |||||||||
43451:      187 cttgagtcgataaccttcatgtagtggaccgggtcgtcatcctctattcttttcccaact 246


HAG002A23R: 179 ccgaactttacttcatcatccccatacctcaaggtgagtgttccgtcattcatatctacc 238
                ||||||||||| ||||| |||||||||||||| || ||||| || |||||||| || ||
43451:      247 ccgaactttacctcatcgtccccatacctcaangttagtgtcccttcattcatgtccact 306


HAG002A23R: 239 actgcttgggcggtggcaaggaagggtctccctagtatgagggggacctcggtgtcttcc 298
                |||||||| |||||||||||||| || |||||||| || |||||||||||| ||||||||
43451:      307 actgcttgtgcggtggcaaggaaaggcctccctagaataaggggggacctcgttgtcttcc 366


HAG002A23R: 299 tccatatcgagtatgacaaagtcggctggatagacgaatctgcttacctttaccaagaca 358
                ||||| |||||||||||||||||  | ||||| || || ||||||||| |||||||||||
43451:      367 tccatgtcgagtatgacaaagtcaacaggataaacaaacctgcttacccttaccaagaca 426


HAG002A23R: 359 ttttcgatgacaccttgtggaaatttgacggatcgatcagcgagttgtatgctcattttt 418
                || |||||||||||||| || || ||||| ||||||||||||||||||||||| ||||||
43451:      427 ttctcgatgacaccttgcgggaacttgaccgatcgatcagcgagttgtatgcttattttt 486


HAG002A23R: 419 gtaaggctcgtggttccccagccaagcctttt 450
                ||| |||||||||||||| |||||||||||||
43451:      487 gtagggctcgtggttcccaagccaagcctttt 518


Query= HAG002B02F
Subject= 44213

HAG002B02F: 1 atagatgacggtaatattcaaactactaaccaaatttatgatgtgtgcgctttgtgtaat 60
              |||||||| |||||| ||||||||||||||||||||||||||||||| ||||||||||||
44213:   3149 atagatgaaggtaatgttcaaactactaaccaaatttatgatgtgtgtgctttgtgtaat 3090


HAG002B02F: 61 gagataggtcacgcggctgaaaattgccaaggaatgttggaagggcaatatgaggaagtc 120
               || |||||||| ||||||||||||||||||||||||||||||||||||||||||||||||
44213:    3089 gaaataggtcangcggctgaaaattgccaaggaatgttggaagggcaatatgaggaagtc 3030
```

```
HAG002B02F: 121 catgcggtccaaggtcaaggccaaggaggaggtggtaggaactacaacaacatgaattct 180
                |||||||| ||||||||||| ||||||||||||||||||||||||||||||||||||||||
44213:     3029 catgcggttcaaggtcaaggtcaaggaggaggtggtaggaactacaacaacatgaattct 2970


HAG002B02F: 181 aatacctaccaccccgggttgaggaatcacccgaactttagatatgggaacccgtcaaat 240
                ||||||||||||||||||||||||||||||||||||||||||||||||||||||||||||
44213:     2969 aatacctaccaccccgggttgaggaatcacccgaactttagatatgggaacccgtcaaat 2910


HAG002B02F: 241 caagcgaacccaaattttcaaggtaaccaaggtaattttggttcacggccatcttataat 300
                ||||||||||||||||||||||||| |||||||||||||||||||||||||||||| |||
44213:     2909 caagcgaacccaaattttcaaggtagccaaggtaattttggttcacggccatcttanaat 2850


HAG002B02F: 301 aaccaaggtgggtaccggggcggaaataaccaagggtatcaaaaacaataccaaacgggt 360
                ||||||||||||||||| ||||||||||||||||||||||||||||||||||||||||||
44213:     2849 aaccaaggtgggtaccgnggcggaaataaccaagggtatcaaaaacaataccaaacgggt 2790


HAG002B02F: 361 caagaacaagggggttcttcgggtggaaatgaggtgatggagatgcttaaaagcatgcaa 420
                ||||| ||| |||| |||||||||||||| ||||||||||||||||| || |||||||||
44213:     2789 caagancaangggggntcttcgggtggaaangaggtgatggagatgctnaanagcatgcaa 2730


HAG002B02F: 421 ttggagatgcaaaagcggaatcaacttgatgaagtgcggatgcaaaaagatgaggttcgc 480
                 |||| |||||||| ||||| ||||||||||||||||| |||||||||||||||||||| 
44213:     2729 ntgganatgcaaaancggaancaacttgatgaagtgcgnatgcaaaaagatgaggttcgn 2670


HAG002B02F: 481 gataaaagcatccattcactaacaacccaaatgggtcaattagcaaccgaggtggcggaa 540
                |||||||||||||| ||||||||||||||||||||||||||||| ||||| ||||| |||
44213:     2669 gataaaagcatccantcactaacaacccaaatgggtcaattagcnaccgangtggcngaa 2610


HAG002B02F: 541 ttgaagaaaggtaagggtcaacttccgagcgacacaaaggtaaacccttcacatggttcg 600
                |||||||||||||||||||||||||| |||||||| ||||||||||||||||||||||||
44213:     2609 ttgaagaaaggtaagggtcaacttccaagcgacactaaggtaaacccttcacatggttcg 2550


HAG002B02F: 601 tcacgaggtaatgttaatattaaccatgttagtgtgttaagaagtgggaaagagtttaag 660
                ||||||||||||||||||||||| ||||||||||| ||||||||||||||||||||||| 
44213:     2549 tcacgaggtaatgttaatattaancatgttagtgtnttaagaagtgggaaagagtttaan 2490


HAG002B02F: 661 ggccatttgtcacccgaattggtcgaaggggtggttgaggacatcacgggaaatggaaag 720
                | | ||||||||||||||||||| || ||||| |||||||| |||||||| |||||||||
44213:     2489 gccaatttgtcacccgaattggtngaggggtngttgagganatcacggg-aatggaaag 2431


HAG002B02F: 721 tgatg 725
                |||||
44213:     2430 tgatg 2426


Query= HAG002C16F
Subject= 44183


HAG002C16F: 1 ttgccaatttggcttgttgcttgaaataggatgaactcaaggcttcatcgccttggtcat 60
              ||||||||||||||||||||||||||||| |||||||| |||||||||||||||||||||
44183:   1588 ttgccaatttggcttgttgcttgaaatagaatgaactcgaggcttcatcgccttggtcat 1529
```

```
HAG002C16F: 61 tcgtgttcgagttatttcctgccacattagttttcattagcttgacccgcagagtccacc 120
               ||||||| |||||||||||||| ||||| |||||||||||||||||||||||||||||||
44183:    1528 tcgtgtttgagttatttcctgctacattggttttcattagcttgacccgcagagtccacc 1469


HAG002C16F: 121 aggatttctgtgcactacaagtcgttattacgtgggcccccgtaataataaattgtcttg 180
                ||||||||||||||||||||||||||||||||||||||||||||||||||||||||||||
44183:     1468 aggatttctgtgcactacaagtcgttattacgtgggcccccgtaataataaattgtcttg 1409


HAG002C16F: 181 cacagttaccccaaattttctctcgtccaagcagatgatcaatcaaggagcaacagcagg 240
                ||||||||||||||||||||||||||||||||||||||||||||||||||||||||||||
44183:     1408 cacagttaccccaaattttctctcgtccaagcagatgatcaatcaaggagcaacagcagg 1349


HAG002C16F: 241 tgcattggcatgaacagggtcatgctctaatgcggtgttaggagcgatgcctggctcagg 300
                ||||| ||||||||||||||||||||||||||||||||||||||||| ||||||||||||
44183:     1348 tgcatcggcatgaacagggtcatgctctaatgcggtgttaggagcgacgcctggctcagg 1289


HAG002C16F: 301 ggccatggctggctccggatcaacaaaccccatctcaaaattagctggatcaaccatcac 360
                ||||| |||||||||||||||||||||| |||||||||||| ||||||||||||||||||
44183:     1288 ggccacggctggctccggatcaacaaactccatctcaaaatcagctggatcaaccatcac 1229


HAG002C16F: 361 aggggttacaggatcctccaagggctggcctaagcgtatgaactcaaggtcatggtctg 420
                ||||||||||||||||||||||||||||||||||||||||||||||||||||||||||||
44183:     1228 aggggttacaggatcctccaagggctggcctaagcgtatgaactcaaggtcatggtctg 1169


HAG002C16F: 421 gatcaaaaccagatagaagaacaggatcaccctcaatactgtgatcaaactcaaagctgt 480
                |||||||||||||| ||||||||||||||||||||||||||||||||||||||||||| |
44183:     1168 gatcaaaaccagatggaagaacaggatcaccctcaatactgtgatcaaactcaaagctat 1109


HAG002C16F: 481 gtgcgggaaacggtgcagctgatgatgccgtatcagggtcggcgtcgtgtgaatggtgct 540
                ||||||||| ||||||||||||||||||||||||||||||||||| ||||||||||||||
44183:     1108 gtgcgggaaccggtgcagctgatgatgccgtatcagggtcggcgtngtgtgaatggtgct 1049


HAG002C16F: 541 gcactccctgtatatgcgaaaatgcggatgtcaggaactcaaatgggtccggaacaggga 600
                |||||||||||||||||||||||||||||||||| |||||||||| ||| || ||||||
44183:     1048 gcactccctgtatatgcgaaaatgcggatgtcagnaactcaaatgagtctgggacagggg 989


HAG002C16F: 601 aatgggcataagtctcccctgcaggagcgtccgcaagcagtagg 644
                ||||||||| ||| ||||||||||||||||||||||||||||||
44183:      988 aatgggcatgagtttcccctgcaggagcgtccgcaagcagtagg 945
```

Query= HAG002C20F
Subject= 65665

```
HAG002C20F: 1 ataatgttcaaaggaacaccatgtcgggatacaatctcatccacatagattttagcaagt 60
              |||||||||||||||||||||||||| |||||||||||||| ||||| || || |||||
65665:   1058 ataatgttcaaaggaacaccatgtcgagatacaatctcatcaacatanatcttggcaagn 999


HAG002C20F: 61 ttgtccgcagatagatccgcacggatcggcaagaaatgtgctgacttagttagatgatcg 120
               | ||| |||||||||||| ||||||||||||||||||||| |||||| || ||| || |
65665:     998 tngtcagcagatagatcctcacggatcggcaagaaatgtgntgactttgtaagacganca 939
```

```
HAG002C20F: 121 acgattacccaaatggcatcatgacctctattagtacgtggcagcttggtaatgacatcc 180
                || |  |||||||| |  ||||||||| | ||||| || || |  |||||||||| ||||
65665:      938 acaacnacccaaatngngtcatgacctntcttagtgcgcggnannttggtaatgagatcc 879


HAG002C20F: 181 atagcaatgttttcccatttccaaaccggtatctctggttgctccaataatccagaggga 240
                |||| |||||||||||||||||||||||| |||| |||||||||||||||||||||||||
65665:      878 atagnaatgttttcccatttccaaaccggnatctntggttgctccaataatccagaggga 819


HAG002C20F: 241 tgttgatgttctgccttaaccttaagacatgtaaggcatttggaaacatataaggcaatg 300
                ||||||||||||||||||||||| ||| | || |||||||| ||||||||||||||||| 
65665:      818 tgttgatgttctgccttaaccttnagataagtnaggcatttngaaacatataaggcaatn 759


HAG002C20F: 301 tctttcttcatacccgaccaccaatactaggttcgaagattcttctacattttatctgca 360
                || ||||||||||||| ||||||||||| ||||||||||| ||| |||||||| ||||||
65665:      758 tcnttcttcatacccggccaccaatactgggttcgaagatccttatacattttgtctgca 699


HAG002C20F: 361 ccaagatgaataaaataacgagacttgtgagcttcatccattagcaaggtgcggagattg 420
                ||| |||||||| ||||||||||||| |||||||||||||| ||||||||||| ||||||
65665:      698 ccaggatgaatagaataacgagacttatgagcttcatccataagcaaggtgcgaagattg 639


HAG002C20F: 421 tcttgacttgggacccacaagcggcccatgaaataatatagcccatagtctttccgttta 480
                |||  ||||||||||||||| ||||||||||||||  | | |||| ||||| || ||| |
65665:      638 tctatacttgggacccacaaacggcccatgaaatagaanatcccaaagtctctcagttca 579


HAG002C20F: 481 agattaggcttaatatgatatggtaattccttacccatcaagtcttgtgtaacaaaagta 540
                || | ||| ||| ||||| | ||||| || |||||||||||||||||||||||| |||||
65665:      578 agctcaggattagtatgacanggtaactctttacccatcaagtcttgtgtaacacaagta 519


HAG002C20F: 541 tgttgagcctgagaaatgcaggtttgaatatcagattgaacctgaacacagtgaagcttg 600
                ||||||||||||   ||||  | |||||| |||||| |||||||||||||||||||||||
65665:      518 tgttgagcctgaattatgcgngattgaatgtcagatcgaacctgaacacagtgaagcttg 459


HAG002C20F: 601 acacgttccttacgactcaatgcgtcagccacgacattcgccttaccaggatgctagcga 660
                ||||||||||||||||| ||||| ||||||||||||||||||||||||||||  ||||||
65665:      458 acacgttccttacgacttaatgcntcagccacgacattcgccttaccaggatagtagcga 399


HAG002C20F: 661 a 661
                |
65665:      398 a 398



Query= HAG002D18F
Subject= 67588

HAG002D18F: 1 tttttgcttttagcacttttgggtgttacatacgttacttatctaaaatcacaatcgaac 60
              |||||||||| |||||||||||||||||||||||||||||||   |||||||||||||||
67588:   1262 tttttgctttaagcacttttgggtgttacatacgttacttattcnaaatcacaatcgaac 1321


HAG002D18F: 61 acactactcaaatatttaaacgctaaccgattcgcatgtattacgtgactaaatgaatgc 120
               ||||||||||| ||||| |||||||||||||| |||||||||||||||||||||||||||
67588:    1322 acactactcaattatttgaacgctaaccgatt-gcatgtattacgtgactaaatgaatgc 1380
```

```
HAG002D18F: 121 ttgttg-ttatgtttacacgtggaatg-ctgtctacctgccttaacgacg-tagtactat 177
                |||||| || |||||||||||||||  |||| |||||||||||||||| |||||||||
67588:     1381 ttgttgattntgtttacacgtggaatgtntgtcnacctgccttaacgacgatagtactat 1440


HAG002D18F: 178 agtttggactcagcacccgttcacacgggggttgttaaggacaattacttttatggatta 237
                ||||||||||||||||||||||||||||||||||||||||||||||||||  ||||||||
67588:     1441 agtttggactcagcacccgttcacacgggggttgttaaggacaattacttgcatggatta 1500


HAG002D18F: 238 tggtggtaatcatgtattgcgaactgtctcggacagtcaacccgtagtcattggtatcga 297
                ||||||||||||||||||||||||||||||||||||||||||| |||||||||||||||
67588:     1501 cggtggtaatcatgtattgcgaactgtctcggacagtcaacccgcagtcattggtatcga 1560


HAG002D18F: 298 tagatccatatcgataattaacatgcttcgttttcctctgtgtacgtgctggttatgcgt 357
                ||||||||| ||||||||||||||||||||||||||||||||||||||||||||||||||
67588:     1561 tagatccatgtcgataattaacatgcttcgttttcctctgtgtacgtgctggttatgcgt 1620


HAG002D18F: 358 aaactatttcaaactctatatgctattatcaaaattgtatgctcacctttacattatatg 417
                |||||||||| |||||||||||||||||||||| ||||||||||||||||||||| ||||
67588:     1621 aaactatttcgaactctatatgctattatcaaacttgtatgctcacctttacattttatg 1680


HAG002D18F: 418 tattgactttattttaacgtatgtgacaggcaattaggatgcttatctgctaggaaggcg 477
                |||||||||||||||||||||||||||||||||||||||||||||||||||||||||||
67588:     1681 tattgactttattttaacgtatgtgacaggcaattaggatgcttatctgctaggaaggca 1740


HAG002D18F: 478 ggctatgaataagcgtctagagcatagttgtctgtagatcttgtcagaccta--gtctct 535
                ||   ||||||||||||||||||||||||||||||||||||| |||| | |  ||||||
67588:     1741 agcntagaataagcgtctagagcatagttgtctgtagatcttg-cagatcnagggtctct 1799


HAG002D18F: 536 agaagcatttgaacagtatttatatttatttaaatctgagttgtcgggacag----aat 591
                ||||||| || ||||  |||  ||   ||||||||||||||||||||| ||||     ||
67588:     1800 agaagcanttaaaca--attgcta---tatttaaatctgagttgtcggaacagttcccat 1854


HAG002D18F: 592 atttgactggttgttatctgtaataatttgttttaccatttgggatacggtatgggacgt 651
                | |||  | |||| | ||| | |||| ||||||    |||||||||||||||||||||||
67588:     1855 a-ttgtttagttgatttctatgataacttgtttatttatttgggatacggtatgggacgt 1913


HAG002D18F: 652 attatttaaactaaatggtaatgatagttgttgtggaaacttctggacaatctgtttcgc 711
                || | ||||   || | ||| | ||||||||| |||||||||||||||||||||||||||
67588:     1914 atcanttaactgaattngtantaatagttgttntggaaacttctggacaatctgtttcgc 1973


HAG002D18F: 712 tc 713
                ||
67588:     1974 tc 1975



Query= HAG002E01F
Subject= 41457

HAG002E01F: 1 acgtccacgaccacgtccatgaccacgaccatgactacgaccatgaccacgcccacgttg 60
              ||||||||||||||||||||||||||||||| ||||||||||||||||||||||||||||
41457:   1817 acgtccacgaccacgtccatgaccacgaccacgactacgaccatgaccacgcccacgttg 1758
```

```
HAG002E01F: 61 atagccacgaccacgtccgtgacttccactttggtcattatatgttgccacattcacttc 120
               ||||||||||||||||||||||||||||||||||||||||||||||||||||||||||||
41457:    1757 atagccacgaccacgtccgtgacttccactttggtcattatatgttgccacattcacttc 1698


HAG002E01F: 121 tgggaatggggttgtaccaaccggacaagtttcatggtttttcatcagtagctcattatt 180
                |||||||||||||||||||||||||| |||||||||||||||||| ||||||||||||||
41457:     1697 tgggaatggggttgtaccaaccggacgagtttcatggtttttcattagtagctcattatt 1638


HAG002E01F: 181 ttgctcagccacaagcaaacatgatattaagtcactgtatttggtgaagcccctttcacg 240
                ||||||||||||||||||||||||||||||||||||||||||||||||||||||||||||
41457:     1637 ttgctcagccacaagcaaacatgatattaagtcactgtatttggtgaagcccctttcacg 1578


HAG002E01F: 241 atattgttgttgcaagacaatgtttgaggcgtgaaaggtggagaatgtttttccaacat 300
                ||||||||||||||||||||||||||||||||||||||||||||||||||||||||||||
41457:     1577 atattgttgttgcaagacaatgtttgaggcgtgaaaggtggagaatgtttttccaacat 1518


HAG002E01F: 301 ctccttatctgtaatattttcaccacatagaatcaattgtgacgtgattctaaacattgc 360
                ||||||||| ||||||||||||||||||||||||||||||||||||||||||||||||||
41457:     1517 ctccttatcagtaatattttcaccacatagaatcaattgtgacgtgattctaaacattgc 1458


HAG002E01F: 361 tgagttatactcacttatagacttaaagtcttgcaaccttaaattaatccattcataacg 420
                ||||||||||||||||||||||||||||||||||||||||||||||||||||||||||||
41457:     1457 tgagttatactcacttatagacttaaagtcttgcaaccttaaattaatccattcataacg 1398


HAG002E01F: 421 agctcttggtaatattactgttttctgatggtcatacctttcttttaaattgatccatag 480
                |||||||||||||||||||||||||||||||||||||||||||||||||||| |||||||
41457:     1397 agctcttggtaatattactgttttctgatggtcatacctttcttttaaattggtccatag 1338


HAG002E01F: 481 aacgagtggatctttgatagtaagatattcattttcaatgactcatgaatatggtggcg 540
                 |||||||||||||||||||| ||||||||||||||||||||||||||||||||||||||
41457:     1337 gacgagtggatctttgatagtgagatattcattttcaatgactcatgaatatggtggcg 1278


HAG002E01F: 541 taagaaaatcattgcctttgctttttcttgggtacttgttttatttccttctttaattgt 600
                |||||||||||||||||||||||| ||||||||||| |||||||||||||||||||||||
41457:     1277 taagaaaatcattgcctttgctttatcttgggtactcgttttatttccttctttaattgt 1218


HAG002E01F: 601 atccccaaggttattggcatttagatggatttcatcatccaaagcccatgataaataatt 660
                |||||||||||||||||||||||||||||||||| |||||||||||||||| ||||||||
41457:     1217 atccccaaggttattggcatttagatggatttcagcatccaaagcccatgacaaataatt 1158


HAG002E01F: 661 ttttccagtgatgtctaaagccataaactcaagctttgcaagattcgacatgattaa 717
                |||||||||||||||||||||||||||||||||||||||||||||||||||||||||
41457:     1157 ttttccagtgatgtctaaagccataaactcaagctttgcaagattcgacatgattaa 1101



Query= HAG002F11F
Subject= 43835

HAG002F11F: 1 aagacgctctactttgccgcgcgaacgtctaaagcaactatgggttcaatcttggcttga 60
              |||||||||||||||||||||||||||||||||||||||||||||| || ||||||||||
43835:    204 aagacgctctactttgccgcgcgaacgtctaaagcaactatgggtttaaacttggcttga 263
```

```
HAG002F11F: 61  tagacaatgggtggttaattccttttaactactcctagacctaccaatatcatcgagcct 120
                |||||||||||  |||||||||||||||||||||||||||||||||||||| ||||||||
43835:      264 tagacaatgggnngttaattccttttaactactcctagacctaccaatatcctcgagcct 323

HAG002F11F: 121 ttccgcgacgagtctagcagcacacttgatttaattaattaccgaatattgttcctaag 180
                ||||||||||||||||||||||||||||||||||||||||||||||||||||||||||||
43835:      324 ttccgcgacgagtctagcagcacacttgatttaattaattaccgaatattgttcctaag 383

HAG002F11F: 181 gttactagtgcacttttcaccctaaagaattaatgacctattatgtgatatagacactca 240
                ||||||||||||||||||||||| ||||||||||||||||||||||||||||||||||||
43835:      384 gttactagtgcacttttcaccctanagaattaatgacctattatgtgatatagacactca 443

HAG002F11F: 241 cctaagccaaaaaccctaattcaactctattccgtgataaagaccgtcaccaagaattga 300
                |||||| |||||||||||||||||||||||||||||||||||||||||||||||||||||
43835:      444 cctaagncaaaaaccctaattcaactctattccgtgataaagaccgtcaccaagaattga 503

HAG002F11F: 301 atggaacaaataaacgattaactaagaaatcacaagcatacaaacgcaccaagacaaact 360
                |||||||||||||||||||||| |||||||||||||||||||||||||||||||||||||
43835:      504 atggaacaaataaacgattaantaagaaatcacaagcatacaaacgcaccaagacaaact 563

HAG002F11F: 361 aacatagtgtttacaatgcaagaaaaatcacaaccacatcaataatcatataaaaatcca 420
                | |||||||||||||||| |||||||||||||||||||||||||||||||||||||||||
43835:      564 ancatagtgtttacaatncaagaaaaatcacaaccacatcaataatcatataaaaatcca 623

HAG002F11F: 421 atcttcgttatgtcataatcatcgcctaggtagtttacgagttttagccggaaaacataa 480
                | ||| || ||| |||| ||||| ||||||||||||| | ||||||||||||||||||||
43835:      624 ancttngtnatgccatantcatcacctaggtagtttatg-gttttagccggaaaacataa 682

HAG002F11F: 481 tcagaaacaaagtcaaaatcaaggtatcaactgaattcatcgttaacaaagtctaaacaa 540
                ||| |||||||||||||| ||  ||||||||||||||||||| |||||||||||||||||
43835:      683 tcaaaaacaaagtcaaaaacatagtatcaactgaattcatcgntaacaaagtctaaacaa 742

HAG002F11F: 541 acaaagaatgcaagaaataggtgtataaaacccgaatcttcactcccgaatgctcaagaa 600
                |||||||||||||||||||||| | || ||||||||||||||||||||||||||||||||
43835:      743 acaaagaatgcaagaaataggtatgtagaacccgaatcttcactcccgaatgctcaagaa 802

HAG002F11F: 601 tgc-ttcccgatgattcctagcttccggtatgcctccgacacgatcacagccttcaatta 659
                ||| ||||||||||||||  ||||||||||||| |||||||||||||||||||||||| |
43835:      803 tgctttcccgatgattccangcttccggtatgcntccgacacgatcacagccttcaatca 862

HAG002F11F: 660 gcagccaaaacagcccc 676
                |||||||||||||||||
43835:      863 gcagccaaaacagcccc 879
```
