# Supplementary material for: The repetitive component of the sunflower genome as shown by different procedures for assembling next generation sequencing reads
Source: BMC Genomics. 2013 Oct 6;14:686. doi: 10.1186/1471-2164-14-686 (PMC3852528; doi:10.1186/1471-2164-14-686)
Supplement: Additional file 2 — The 5 sets of CLC-BIO parameters used for mapping of the Illumina reads to 60 sunflower DNA sequences with known redundancy ([20]; Giordani, personal communication) and correlation between known copy number and average coverage. [file 1471-2164-14-686-S2.pdf]

Additional file 2. The 5 sets of CLC-BIO parameters used for mapping of the Illumina reads to 60 sunflower DNA sequences with known redundancy, and correlation between known copy number and average coverage

**Set**	**Parameters**					**Correlation (r^2)**	**Significance (*P*)**
	**Mismatch Cost**	**Deletion Cost**	**Insertion Cost**	**Length fraction**	**Similarity**		
1	1	1	1	0.7	0.7	0.7803	<0.0001
2	1	1	1	0.8	0.8	0.7757	<0.0001
3	1	1	1	0.9	0.9	0.7872	<0.0001
4	2	2	2	0.9	0.9	0.7869	<0.0001
5	3	3	3	0.9	0.9	0.7852	<0.0001
